# Prevalence, awareness and control of hypertension in Ghana: A systematic review and meta-analysis

**DOI:** 10.1371/journal.pone.0248137

**Published:** 2021-03-05

**Authors:** William Kofi Bosu, Dary Kojo Bosu

**Affiliations:** 1 Department of Public Health and Research, West African Health Organisation, Bobo-Dioulasso, Burkina Faso; 2 Department of Paediatrics, St Dominic’s Hospital, Akwatia, Ghana; University of Perugia, ITALY

## Abstract

**Background:**

Hypertension is a major health problem in Ghana, being a leading cause of admissions and deaths in the country. In the context of a changing food and health policy environment, we undertook a systematic review (PROSPERO registration number: CRD42020177174) and a meta-analysis of the prevalence of adult hypertension, and its awareness and control in Ghana.

**Methods:**

We searched major databases including PubMed, Embase as well as Google Scholar and online digital collections of public universities of Ghana to locate relevant published and unpublished community-based articles up till April 2020.

**Findings:**

Eighty-five articles involving 82,045 apparently-healthy subjects aged 15–100 years were analyzed. In individual studies, the prevalence of hypertension, defined in most cases as blood pressure ≥ 140/90 mmHg, ranged from 2.8% to 67.5%. The pooled prevalence from the meta-analysis was 27.0% (95% CI 24.0%-30.0%), being twice as high in the coastal (28%, 95% CI: 24.0%-31.0%) and middle geo-ecological belts (29%, 95% CI: 25.0%-33.0%) as in the northern belt (13%, 95% CI: 7.0%-21.0%). The prevalence was similar by sex, urban-rural residence or peer-review status of the included studies. It did not appear to vary over the study year period 1976–2019. Of the subjects with hypertension, only 35% (95% CI: 29.0%-41.0%) were aware of it, 22% (95% CI: 16.0%-29.0%) were on treatment and 6.0% (95% CI: 3.0%-10.0%) had their blood pressure controlled. Sensitivity analyses corroborated the robust estimates. There was, however, high heterogeneity (I^2^ = 98.7%) across the studies which was partly explained by prevalent obesity in the subjects.

**Conclusion:**

More than one in four adults in Ghana have hypertension. This high prevalence has persisted for decades and is similar in rural and urban populations. With the low awareness and poor control of hypertension, greater investments in cardiovascular health are required if Ghana is to meet the global target for hypertension.

## Introduction

Hypertension is among the leading causes of admissions and deaths in Ghana [1, 2]. It was the third leading cause of admission and the leading cause of deaths, accounting for 4.7% of the total admissions and 15.3% of the total deaths in Ghana in 2017 [1]. Patients are admitted for one to 91 days, with 22.7% staying for four or more days [2]. The outpatient burden of hypertension has been increasing. In one region, the number of new cases increased 3.8-fold in five years from 35,855 in 2006 to 138,040 in 2010 [3].

Hypertension is a common cause of medical emergencies such as heart failure [4] and renal failure [5] in Ghana. It is the main determinant of stroke in Ghana, with a population attributable risk of about 91% [6]. Among the elderly patients aged >60 years involved in a prospective study in Ghana, the risk of incident stroke increased with increasing levels of blood pressure (BP) with 0 stroke events/100py for BP <120/80 mmHg, 1.98 (95%CI: 1.26–2.98) for BP 120-159/80-99 mmHg and 2.46 events/100py (95% CI: 1.20–4.52) at BP >160/100 mmHg [7].

Besides genetic factors, behavioural risk factors such as eating foods high in salt and fat, inadequate intake of fruit and vegetables, harmful use of alcohol use, tobacco smoking, low physical activity and poor stress management contribute to the development of hypertension [8]. Older age and overweight/obesity appear to be consistent determinants of hypertension in Africa [9]. Analysis of household survey data in five sub-Saharan Africa countries (Benin, Burundi, Ghana, Kenya and Lesotho) showed that women with overweight or obesity were 2.4 and 5.3 times as likely as those with normal body mass index (BMI) to have hypertension [10]. Overweight/obesity, physical inactivity, older age and family history of diabetes are also significantly associated with adult diabetes in Ghana [11].

In 2010, we published a systematic review of studies involving 26,649 adult participants which revealed a prevalence of hypertension ranging from 19% to 48%, with higher prevalence in urban centres, in the national capital and in older subjects [12]. Since then, the Government of Ghana has developed national strategic documents on noncommunicable diseases (NCDs) and nutrition that seek to reduce the burden of hypertension in the country [13, 14]. In 2019, a draft revised national NCD policy of Ghana was prepared whose goal is to reduce the burden of NCDs is to the barest minimum to render it of little or no public health importance [15].

Besides the health policy environment, the risk factor profile in Ghana has been changing in favour of obesity. Ghana is one of the few Sub-Saharan Africa (SSA) countries undergoing nutrition transition, along with South Africa, Cabo Verde and Senegal, as assessed by 18 indicators [16]. The prevalence of women with overweight and obesity in women is not only among the highest in SSA [10], but increased significantly in surveys conducted between 1991 to 2014 [17]. A meta-analysis published in 2016 estimated that 42.5% of adults in Ghana lived with overweight or obesity [18]. Energy-dense foods, including fried and processed foods, fast food along with sugar-sweetened beverages are widely available on the streets in urban centres [19, 20]. Both rural and urban dwellers prefer to dine outside their homes as their incomes improve [21].

While we did not directly assess their effects on hypertension, we thought the changing health policy and nutrition landscape warranted conducting another systematic review on adult hypertension in Ghana. Unlike the previous review, we sought to estimate the pooled prevalence of adult hypertension as well as pooled estimates of the level of its awareness, treatment and control. We also assessed the severity of adult hypertension from reported studies in Ghana. We expect our findings to inform the national policy on the burden of hypertension in Ghana as well as guide the country in monitoring progress towards the achievement of the NCD global targets by 2025 [22].

## Methods

The study protocol is registered in the international prospective register of systematic review, PROSPERO (CRD42020177174). The conduct and reporting of the systematic review and meta-analysis has been guided by the Preferred Reporting Items for Systematic Reviews and Meta-Analyses (PRISMA; S1 Table) [23].

### Search strategy, inclusion and exclusion criteria

We searched the major databases, Ovid Medline, Ovid Embase, Academic Search Ultimate, CINAHL, PsychInfo and Web of Science as well as the African Journal Online (AJOL) repository for articles published until 7 April 2020. For grey literature, we searched ProQuest, and Google Scholar. Unlike our earlier review, we also searched the recently-available repositories with digital collections of students’ dissertations of three public universities—the University of Ghana Digital Collections (UGSpace), the Kwame Nkrumah University of Science and Technology (KNUSTSpace), University of Development Studies (UDSpace).

Our search strategy used comprehensive search terms (hypertension, blood pressure, prevalence, proportion, incidence, Ghana), guided by a modified participants-intervention-comparison-outcome-context (PICOC) framework, since there was no comparison group or intervention [24]. In order to maximize the yield of articles, the results from the different stages of the search process were combined by appropriate Boolean operators, ‘OR’ and ‘AND’ (S2 Table). There were no language restrictions. We restricted the articles to studies conducted in “humans” in the age groups of adolescents (13–18 years) and adults (19 years and older). While the focus was on adult hypertension, we included the pre-specified adolescent age group in the filter to ensure that studies among adults in which the lowest age group was 13 years or older were captured. The bibliographies of retrieved articles were manually-searched to locate additional articles.

Included studies were those that were community-based, cross-sectional or follow-up in design, conducted among apparently healthy adult subjects living in Ghana, and provided an estimate of the prevalence of hypertension. Adult hypertension was defined using the BP ≥140/90 mmHg cut off or those taking anti-hypertensive treatment regardless of their blood pressure on measurement [25]. Studies reporting the prevalence of either systolic (BP >140 mmHg) or diastolic (DBP >90 mmHg) hypertension or both were included. Where systolic and diastolic hypertension were separately provided, we selected the higher of the two for analysis. In one study in which adolescents were included with the adult sample [26], hypertension in adolescents was defined as having SBP or DBP greater than or equal to the 95^th^ percentile in line with the recommendations of the National High Blood Pressure Education Program Working Group on High Blood Pressure in Children and Adolescents [27].

Multi-country studies were included if data on the prevalence of hypertension in Ghana could be distinctly extracted. Conference proceedings, abstracts, correspondence or editorials which contained reported the prevalence of adult hypertension were included.

Studies conducted exclusively among adolescents were excluded as were those involving hospital-based patients, overtly sick subjects (for example, those in hospices), migrants or participants not living in Ghana and pregnant women were excluded. Participants with non-systemic hypertension, self-reported hypertension or with hypertension defined based on a different BP cut-off were also excluded.

### Study selection

The citations obtained from the different databases were imported into Covidence, a software for the management of systematic reviews [28]. Articles from the AJOL, Google Scholar, ProQuest and the university digital collections could not be imported directly into Covidence and so were screened manually. Covidence removed duplicates and managed the remaining steps in the study selection. The titles and abstracts were screened for relevance and those deemed not eligible excluded. The full-text version of potentially eligible articles was retrieved and screened for conformity to the inclusion criteria. The reasons for excluding any full-text articles were documented. Where there were several publications from the same study, one of them was selected as the primary study while all the secondary publications were excluded to avoid multiple counts of the same study. The secondary publications frequently involved sub-groups based on sex or age group. The primary study selected was one that involved the study’s original entire sample or one that contained the most complete data. Occasionally, data such as the sampling procedure, BP measurement protocol and mean BP were extracted from multiple publications of the same study, when the data to be extracted were not all available from a single publication. The screening and selection of articles were done independently by the authors (WKB, DKB), with the aid of the Covidence software, with any disagreements resolved through mutual consensus.

### Data extraction

Using an adapted extraction form from previous systematic reviews on hypertension [12, 29, 30], we (WKB, DKB) extracted a large amount of data including the publication characteristics (title, author, year, external collaboration), study objective, study design, study year, study setting, sample size, sampling procedure, demographics, socioeconomic characteristics, lifestyle factors, blood pressure measurement methods, prevalence of hypertension and its severity awareness, treatment and control. We contacted authors of studies with incomplete or ambiguous information, at least once, often in relation to the study year, the sampling process or the definition of hypertension.

Respondents who reported having previously been diagnosed as having hypertension by a health professional were considered to be aware of their disease condition; those reporting current treatment with anti-hypertensive medication were considered to be on treatment while those whose BP was less than 140/90 mmHg were considered to have their hypertension controlled.

### Quality assessment

The quality of the included studies was independently assessed by two reviewers (WKB, DKB) using a validated tool for prevalence studies [31]. The tool comprises ten questions which assess external validity issues (such as the representativeness of the sample, participation rate, and the sampling technique) as well as internal validity issues (such as the suitability of case definition, reliability of study instrument, and the application of same measurement methods for all subjects). Based on these criteria, each analysed study was classified as having low, moderate or high risk of bias [31]. Disagreements in the assessment were resolved by consensus.

### Data analysis

The prevalence of hypertension across the included studies was pooled together through an inverse-variance weighted random effects meta-analysis with variance stabilization via the Freeman-Tukey double arcsine method [32]. Heterogeneity between the studies was assessed using the Cochran’s Q chi-squared test (alpha set at 0.1) statistic [33] and the Higgins and Thompson’s *I*^*2*^ statistic [34]. The cut-off *I*^2^ values of 0%, 25%, 50%, and 75% represented no, low, moderate, and high heterogeneity, respectively. The individual prevalence estimates from studies as well as the pooled estimates along with their 95% interval estimates were presented in forest plots.

We performed sub-group analysis by sex, geographical belt of residence (coastal, middle and northern), urban-rural residence, publication type, peer-review status of articles, sample size, age of subjects, frequency of BP readings and the definition used for hypertension to identify potential sources of heterogeneity. Studies published in scientific journals as original articles or abstracts were considered peer-reviewed while unpublished university dissertations and study reports were considered non-peer-reviewed. We further explored potential sources of heterogeneity through univariate and multivariate meta-regression analyses with restricted maximum likelihood estimation using study year, publication, percentage obesity, number of subjects screened for hypertension and BP device type as covariates. We checked that there were at least ten studies available for each variable in the model for the analysis [35].

Sensitivity analyses were performed by assessing the effect of eliminating studies deemed to be high risk of bias on the pooled estimate as well as by assessing the effect of removing one study at a time on the pooled estimate in an influence analysis [36]. The presence of reporting bias was evaluated when there were more than ten studies through funnel plot asymmetry and Egger’s test [37]. Except for the leave-one-out influence analysis performed with OpenMeta (analyst) software [38], all statistical analyses were performed in Stata version 15 for windows [39]. Statistical significance was set at the 5% level.

## Results

### Study selection

**Fig 1:**
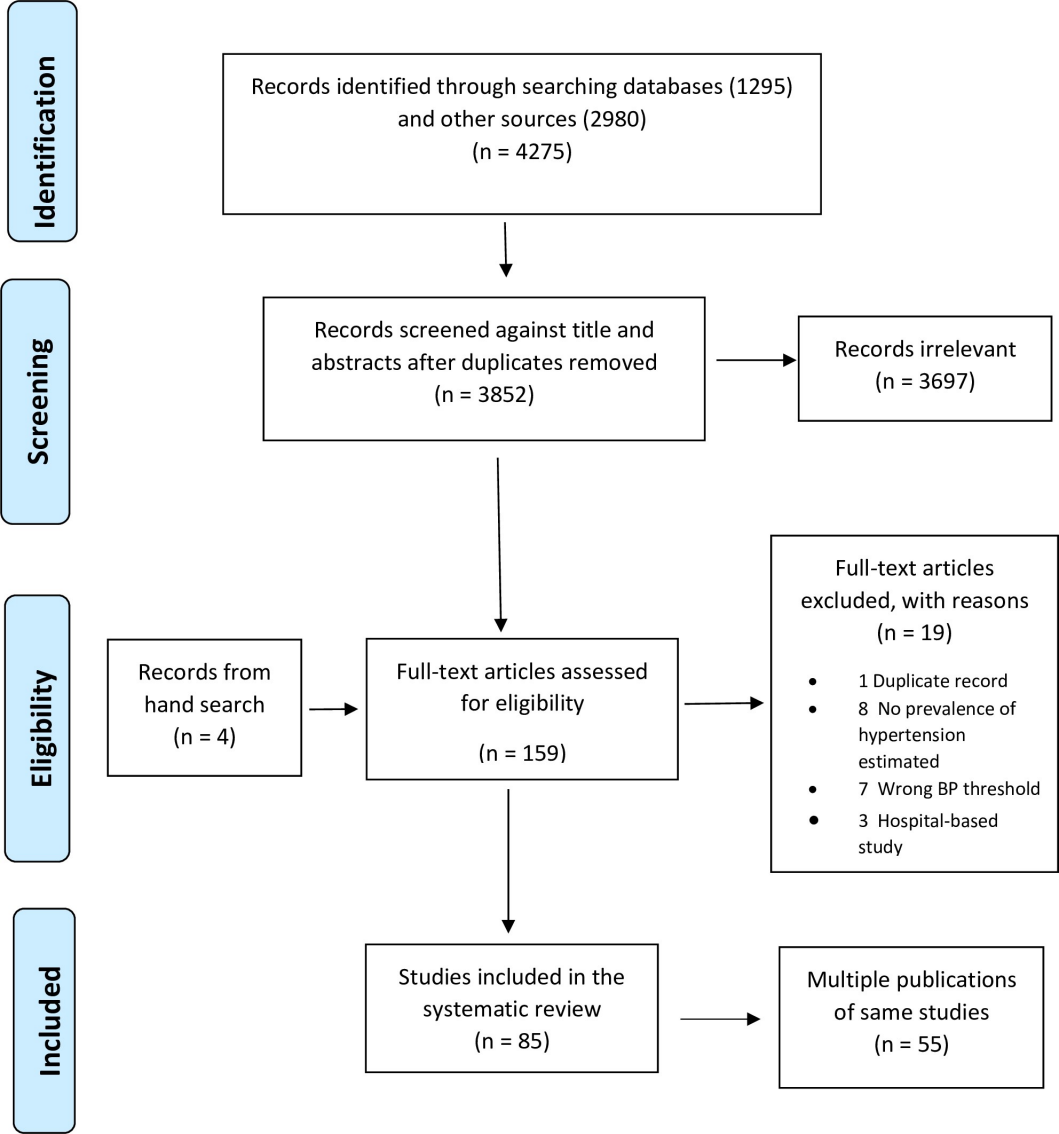
Flow diagram showing study selection.

The study selection process is presented in a PRISMA flow chart (Fig 1) [23]. We identified 1,295 records from the major electronic databases and 4,275 from other sources including 814 from African Journals Online repository, 790 from university digital collections, 980 from Google Scholar and 396 from ProQuest. After excluding 465 duplicate records, we screened 3,852 titles and abstracts out of which we retrieved 155 articles for full-text screening. We excluded 19 studies because they were a duplicate record or did not meet the inclusion criteria. They had no prevalence of hypertension estimated, used wrong non-standard threshold used to define hypertension, or subjects were hospital-based patients. We also excluded 55 were multiple publications of the same study (S3 Table) and identified four additional articles from screening the bibliography of eligible studies. Thus, we finally included 85 articles in the systematic review and meta-analysis.

The highest number of multiple publications were from studies with publicly available datasets such as the first wave of the Study on Global AGEing and adult health (SAGE W1) and the Ghana Demographic and Health Survey (GDHS) with 17 and seven multiple publications besides the studies we designated as their primary publications. These multiple publications were frequently from the analyses of sex, residence or age-based sub-groups of the original sample, or from the use of different definition of hypertension such as systolic hypertension as the outcome variable. Other multiple publications were due to presentation of study results as conference abstracts; analysis of the Ghana sample with that of other countries in multicountry studies; or from analysis of other thematic areas of the same study. There were four dissertations that had also been published in peer-reviewed journals [40–43].

### Study characteristics

Out of the 85 unique articles retained, 65 (76.5%) were single one-off publications, 64 (77.6%) had lead authors based in Ghana and 25 (29.4%) involved external collaborations with researchers in Europe, North America or Asia. A further four (4.7%) studies were published entirely by researchers based outside of Ghana with no local authorship. All the studies were published in the English language.

The studies comprised 61 (71.8%) original articles, 20 dissertations (23.5%), three (3.5%) abstracts and one (1.2%) unpublished report. The earliest study was conducted in 1976 [44] and the latest in 2019 [45–48] (Table 1). The number of studies conducted increased rapidly from two (2.5%) in the pre-2000 years to 22 (27.2%) in the 2000–2009 decade and then to 57 (70.4%) the following decade, 2010–2019. The publication year ranged from 2003 to 2020. The number of articles published increased nearly five-fold from 14 (16.5%) in the 2000–2009 decade to 68 (80.0%) in the 2010–2019 decade. In the modal year 2018, 15 (17.6%) articles were published. Three articles were published during the first four months of the year 2020. The median study year and publication year were 2014 and 2015 respectively.

**Table 1:** Background characteristics of individual studies.

Primary Reference	Study year	Region	Location	Residence type	Participants	Response rate	Mean age ± sd (years)	% with obesity	Number screened for hypertension			% hypertension on BP measurement			% hypertension on BP measurement and medical history			Overall risk of bias
									M	F	T	M	F	T	M	F	T	
Abban 2013 [55]	2012	CR	Cape Coast	Urban	workers		40.78 ± 8.26	14.1	170	0	170			22.4				Low
Abubakari 2018 [56]	2018	GAR	Accra	Urban	workers	100		13.7	113	187	300	8.0	7.0	7.3				Low
Acheampong 2018 [57]	2017	GAR	Oyibi	Urban	students	100	28.3 ± 3.5	16.0	111	76	187	8.1	10.5	9.1				Low
Acheampong 2019 [58]	2017	GAR	Kpone-Katamanso district	Urban	general population	72.0	49.6 ± 13.3	32.4	0	216	216			33.8				Low
Addo 2006 [40]	2001	GAR	Amasaman sub-district	Rural	general population	99.7	42.4 ± 18.6	10.2	107	255	362	24.3	25.9	25.4				Low
Addo 2008 [59]	2006	GAR	Accra	Urban	workers	82.7	44.0 ± 10.1	20	615	400	1015				31.7	28.0	30.2	Low
Adusei 2020 [41]	2015	GAR	Agbobloshie	Urban	workers		25.3 ± 7.5		112	0	112			9.8				Low
Agyapong 2018 [60]	2016	AR	Kumasi	Urban	other			6.3	150	10	160	55.3	90.0	57.5				Low
Agyei-Baffour 2018 [61]	2014	Ahafo	Hwidiem and Nkaseim sub-districts	Rural	general population		39 ± 14.5		249	257	506			21.3				Low
Agyemang 2006 [62]	2004	AR	Kumasi and 4 villages	Mixed	other	82%-99%	35.9	7.2	644	787	1431				31.1	28.1	29.4	Moderate
Aidoo 2015 [63]	2012	GAR	Accra	Urban	workers	40.3	45.1 ± 9.6	64.0	112	49	161			60.2				High
Akufo 2008 [64]	2008	AR	Kumasi	Urban	workers	100	34.5		251	49	300	22.7	18.4	22.0				Low
Amidu 2012 [65]	2009	AR	Bantama; Sofoline	Urban	general population		30.2 ± 7.8	2	200	0	200			12.0				High
Amidu 2016 [66]	2014	NR	Tamale	Urban	general population		34.5 ± 12.3	18.3	133	167	300	15.8	14.4	15.0				Moderate
Amidu 2018 [67]	2009	AR	Atonsu, Meduma, Sofoline, Adum	Urban	general population		35.21 ± 12.87		110	116	226	33.6	37.1	35.4				High
Amoah 2003a [68]	1998	GAR	Labone, Cantonments-Teshie, Danfa, Abokobi	Mixed	general population	75.1	44.3 ± 14.7	14.1	1860	2873	4733						28.4	Low
Amoah 2003b [69]		GAR	Accra	Urban	general population		41.2 ± 1.3		87	113	200	26.4	26.5	26.5				Moderate
Amponsah 2019 [70]	2018	GAR	Agbobloshie	Urban	workers	100.0	26 ± 6.4		28	72	100			19.0				High
Amponsem-Boateng 2017 [71]	2016	AR	AR	Urban	workers		45.4 ± 9.9		182	18	200						67.5	Low
Anderson 2017 [53]	2016	Multi-region	Takoradi; Cape Coast	Urban	general population		46.25 ± 17.14				975			27.0				High
Anto 2020 [54]	2016	Multi-region	Accra, Kumasi	Urban	workers		44.1 ± 9.3	19.0	527		527	38.7		38.7				Low
Aryeetey 2011 [43]	2009	GAR	Accra	Urban	workers	78.3	40.5 ± 10.8	12.8	95	46	141				40.0	21.7	34.0	Low
Atibila 2018 [42]	2015	Bono	Dormaa Ahenkro	Mixed	general population		50.06	4.7	202	198	400	44.6	35.4	40.0				Low
Atinyi 2017 [72]	2017	VR	Keta	Mixed	general population			31.4	72	192	264	58.3	54.2				61.7	Low
Awuah 2014 [73]	2011	GAR	James Town, Ussher Town, Ga Mashie	Urban	general population		31.0 ± 10.6		329	385	714				31.6	25.5	28.3	Low
Basu 2013 [49]	2008	All regions	National	Mixed	general population	99.8					5563						40.9	Low
Bawah 2019 [45]	2019	VR	Ho	Urban	general population			26.2	62	140	202			26.2				Moderate
Bosu 2010 [74]	2006	GAR	GAR	Mixed	general population	99.9	40.4 ± 11.0	26.9	889	1708	2597				41.6	37.9	39.2	Low
Burket 2006 [75]	2002	VR	Liati and Tokor villages	Rural	general population		41.8	9.2	66	218	284	39.4	30.7	32.7				High
Cappuccio 2004 [76]	2002	AR	Kumasi, Ejisu-Juaben	Mixed	general population	53.4	54.6 ± 11.1		385	628	1013				29.9	28.0	28.7	Moderate
Cook-Huynh 2012 [77]	2010	AR	Adankwame	Rural	general population		52.0 ± 19.2		94	232	326			35.0				Moderate
Darko 2012 [78]	2009	GAR	Accra	Urban	general population	91.7		37.46	0	2797	2797			27.2				Low
Donkor 2015 [79]		GAR	Ashaiman Municipality	Urban	general population	100.0		9.8	0	254	254		20.1	20.1				Low
Dosoo 2019 [80]	2015	BER	Kintampo	Mixed	general population				1009	1546	2555						28.1	Low
Duah 2013 [26]	2007	AR	Adansi South district	Rural	general population						442			30.8				Low
Duda 2007 [81]	2003	GAR	Accra	Urban	general population	41.8	46.8 ± 18.0	34.6		1303	1303			54.6				Low
Egungwu 2015 [82]	2015	GAR	Accra	Urban	workers	96.2	33.4	13.0	22	178	200	9.1	20.8	19.5				Low
Ellahi 2017 [83]	2017	VR	Hohoe municipality	Mixed	general population		47.26 ± 16.13		354	496	850			36.0				Low
Escalona 2004 [84]	2002	GAR	Accra	Urban	general population			17.2	257	341	598	27.6	26.1	26.8				High
Frimpong 2018 [85]	2018	GAR	Agbobloshie	Urban	other	100.0	27 ± 8.0		72	28	100			53.0				High
Gato 2019 [86]		CR	Cape Coast	Urban	general population		38.2 ± 10.2	10.0	42	28	70			25.7				High
Gómez-Olivé 2017 [87]	2016	UER	Navrongo	Rural	general population		51.02 ± 5.74		917	1071	1988				24.1	24.8	24.5	Low
Gyamfi 2010 [88]	2010	AR	Sekyere West district	Mixed	general population			10.7	150	150	300	56.7	41.3	49.0				Low
Gyamfi 2015 [89]	2015	AR	Juaben	Urban	workers			60.0	0	50	50			38.0				High
Gyamfi 2018 [90]	2016	AR	Kumasi	Mixed	students	100		5.4	325	215	540	2.8	1.4	2.2				Low
Hayibor 2010 [91]	2010	GAR	Legon	Urban	workers			13.1	265	118	383				30.6	15.3	25.8	Low
Jaziri 2016 [92]	2015	AR	Barekese	Rural	general population			10.1	196	649	845			30.7				Low
Kasu 2015 [93]	2013	OR	Kadjebi district	Mixed	workers	74.5	34.4	12.7	73	85	158	8.2	5.9	7.0				Low
Kodaman 2016 [94]		Bono	Sunyani city & 31 surrounding villages	Mixed	general population		43.05	13.1	1441	1876	3317				29.4	28.6	28.9	Moderate
Koopman 2012 [95]	2010	UER	Garu-Tempane District	Rural	general population	85.4			480	444	924	25.6	22.5	24.1				Low
Kpormegbe 2019 [46]	2019	GAR	Accra	Urban	workers	100.0	35.5 ± 8.0	28.0	104	196	300	22.1	32.7	29.0				Moderate
Kubuga 2015 [96]	2014	NR	Tamale	Urban	workers				74	172	246			4.1				Can’t tell
Kunutsor 2009 [97]	2007	UER	Kassena-Nankana district	Rural	general population	95.7	37.75 ± 14.05		207	367	574			19.3				Low
Lamptey 2017 [98]	2015	ER	LMK district; Akuapem South and Nsawam-Adoagyiri Municipalities	Urban	general population		49	18.9	841	1496	2337				33.1	32.0	32.4	Low
Mensah 2013 [99]	2013	GAR	Accra	Urban	workers			30.8			156			26.3				Moderate
Mensa-Wilmot 2003 [100]	2003	UER	Kassena-Nankana district	Rural	general population	93.1	36.0 ± 14.6	2.7	842	1140	1982	7.2	5.4	6.2				Low
Menyanu 2017 [50]	2015	All regions	National	Mixed	general population	70.0					4675						30.9	Low
Mohammed 2016 [101]	2008	GAR	Accra	Urban	general population	100.0	33.0 ± 7	14.0	90	86	176	32.2	24.4	28.4				Low
Murray 2018 [102]	2016	OR	Nkonya-Wurupong	Urban	general population				92	199	303	18.5	19.6	18.5				High
Newlove 2011 [103]	2007	AR	Obuasi	Urban	general population			22.2	320		320			41.6				Low
Nuertey 2017 [51]	2014	All regions	Ghana	Mixed	other	93.0	67.2 ± 5.4	16.3			4439			47.8				Moderate
Nunoo 2018 [104]	2018	GAR	Accra	Urban	general population	100	77% aged 18–39 yrs	18.0	67	83	150	19.4	19.3	19.3				High
Nyarko 2018 [105]	2018	Bono	Sunyani	Urban	general population				122	221	343	20.5	24.0	22.7				Low
Obirikorang 2015 [106]	2013	AR	Kumasi; Jachie-Pramso	Mixed	general population			36.2	312	360	672	46.2	25.0	34.8				Low
Ofosuhene 2020 [47]	2019	GAR	GAR	Urban	workers	100.0		18.0	186	158	344			7.8				Low
Osei-Yeboah 2018 [107]	2016	WNR	Sefwi-Wiawso	Urban	workers	50	32.1 ± 8.9	12.5	48	64	112	22.9	10.9	16.1				Moderate
Osman 2017 [108]	2016	AR	Kumasi metropolis, AN, EJ, AAC municipality	Mixed	general population	100.0	74.4	16.0	135	265	400	57.8	52.5	54.3				High
Owiredu 2008 [109]	2005	AR	Santasi, Bantama, Old Tafo—in Kumasi	Urban	general population		41.64 ± 13.4	20.4	117	266	383	12.0	29.3	24.0				Moderate
Owiredu 2011 [110]	2010	AR	Kumasi	Urban	other		43.56 ± 1.06				186			16.7				High
Owusu-Sekyere 2018 [111]	2018	GAR	Agbobloshie	Urban	workers	100.0		13.3	91	29	120			35.8				Moderate
Pobee 2006 [44]	1976	GAR	Mamprobi	Urban	general population	73			2001	2702	4703	28.5	23.3	25.5				Low
Pobee 2013 [112]		GAR	Accra	Urban	workers		42.3 ± 6.0	27.0		400	400			11.5				Moderate
Rajaee 2015 [113]	2011	UER	Kejetia; Gorogo	Rural	general population		31.4 ± 10.9	3.5	83	88	171			19.3				Low
Sanuade 2018 [52]	2014	All regions	National	Mixed	general population	99.0	29.6 ± 9.8		3855	9392	13247	12.1	13.4	13.0				Moderate
Sarfo-Kantanka 2014 [114]	2011	AR	Kumasi	Urban	general population			12.0	506	773	1279	32.2	27.9	29.6				High
Sarkodie 2018 [115]	2018	AR	Kwabre East district	Rural	general population						329			35.3				Low
Setorglo 2019 [48]	2019	CR	Abura	Urban	workers	100	36.65 ± 9.76	10.5	200		200			23.0				Low
Shaidah 2016 [116]	2016	GAR	Legon	Urban	workers	100	43.0 ± 10.8	10.9			188			45.2				High
Solomon 2017 [117]	2017	VR	Hohoe	Mixed	general population		40.4 ± 14.5	9.1	180	170	350						39.4	Low
Taylor 2015 [118]	2015	GAR	Accra	Urban	workers	100.0	41.0 ± 11	19.4	50	22	72	14.0	9.1	12.5				High
Vuvor 2011 [119]	2008	GAR	Accra	Urban	general population				288	309	597			17.3				Low
Vuvor 2016 [120]	2015	GAR	GAR community	Urban	general population	20.2	40.0 ± 14.3		207	0	207	26.1		26.1				High
Vuvor 2017 [121]	2016	WR	Shama	Urban	general population			7.5	103	97	200	34.0	30.9	32.5				High
Yakong 2015 [122]	2013	NR	Tamale	Urban	workers	90.9	35.0 ± 9.7	25.0	100	100	200	5.0	1.0	3.0				Low
Yeboah 2015 [123]	2014	ER	Asesewa	Urban	general population			8.8	69	56	125	14.5	23.2	18.4				High

Notes: AAC = Asante Akim Central Municipality, AN = Atwima Nwabiagya District, EJ = Ejisu Juaben Municipality, LMK = Lower Manya Krobo, M = males, F = females, T = total (both sexes); sd = standard deviation

**Fig 2:**
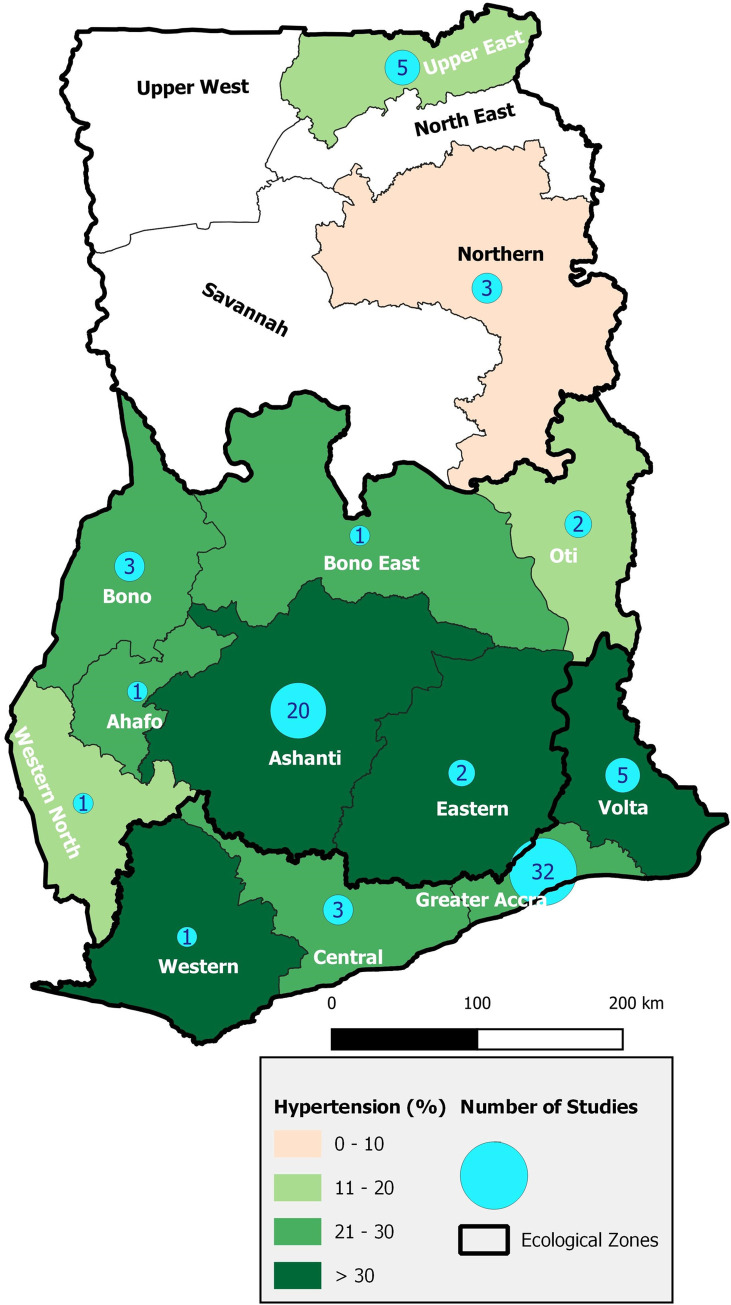
Map of Ghana showing regional distribution of number of studies and the prevalence of hypertension.

Fifty-two (61.2%) studies were conducted either in the Greater Accra Region (37.6%) or in the Ashanti Region (23.5%) (Table 1). The sub-national studies were conducted in 13 (81.2%) out of Ghana’s 16 regions, with no identified studies in the North East, Savannah, and Upper West Regions (Fig 2). However, there were four studies, including the Ghana Demographic and Health Survey (GDHS) and two waves of SAGE, that were nationwide in coverage [49–52]. The regional coverage of the studies translated into 42 (49.4%) studies in the coastal geographical ecological belt, 30 (35.3%) in the middle belt and eight (9.4%) in the northern belt. The coastal belt studies included one which was conducted in two regions [53]. One other study was conducted in two regions, one in the coastal and the other in the middle belt [54]. Fifty-four studies (63.5%) were conducted in urban populations, 12 (14.1%) in rural populations and the remaining 19 (22.4%) in rural populations.

Twenty-seven studies (31.8%) were conducted among occupational workers comprising professional grade workers (e.g. civil servants, health workers, teachers, pastors, bankers, media workers), skilled workers (e.g. factory workers, automobile garage workers, e-waste recyclers, miners, bus or taxi drivers), uniformed personnel (firefighters, security officers), and unskilled workers (e.g. market men and women). The majority of the studies (62.4%) involved the general population while two (2.4%) conducted were among tertiary-level students [57, 90].

The studies were cross-sectional in design; four were panel surveys (cross-sectional time-series)–these being, Waves I and II of the Women’s Health Study of Accra [78, 81] and of SAGE Ghana [49, 50]. No study reported incident hypertension. The response rate among eligible population was infrequently reported. Out of 38 (44.7%) studies with this information, 33 (86.8%) reported response rates of 70%-100%. In the others, the response rate was 20%-53% [63, 76, 81, 107, 120].

A total of 84,184 subjects aged ≥15 years were covered by the 85 studies, with the sample size in individual studies ranging from 50 to 13,247, a median of 320 and mean of 990. In 77 studies with sex-disaggregated data, there were a total of 30,470 men with the number in individual studies ranging from zero to 3,855, a median of 170 and mean of 396. In comparison, there were a total of 47,028 women with their number in the individual studies ranging from zero to 9,392, a median of 198 and mean of 611.

Seven (8.2%) studies were conducted exclusively in men [41, 48, 54, 55, 65, 103, 120] and six (7.1%) exclusively in women [58, 78, 79, 81, 89, 112]. In 69 studies that enrolled both sexes, the proportion of females enrolled ranged from 6.3% to 89.0% with a median of 55.3%.

The age range of the enrolled subjects was provided by only about half (51.8%) of the studies with the extremes being 13->60 years and 65–100 years. The widest age tranche enrolled per study was 18–100 years while the narrowest was 40–75 years. Many studies did not routinely report the mean or median age of the sample. In studies that did not specifically report the mean or median age of participants, there was still sufficient information available to conclusively determine that the age profile satisfied the inclusion criteria. In 53 (62.4%) studies with available information, the mean ages were in the 20s (25.3–29.6 years) in five (9.4%), 30s (30.2–39.0 years) in 18 (34.0%), 40s (44.0–49.6 years) in 24 (45.3%), in the 50s (50.1–54.6 years) in four (7.5%) and ≥60 years in two (3.8%). The reported extreme mean ages were 25.3 ± 75 years among informal workers involved in electronic waste processing activities [41] and 74.4 years among a community-dwelling population [108]. In 72 (84.7%) studies, we determined that the median age was <50 years in 64 (88.9%) and ≥50 years in the other eight (11.1%).

Overall, the studies involved mostly literate populations. In 53 (62.4%) studies with available information, it was only in three (5.7%) in which more than half (51%-68%) of the subjects studied had no formal education. In 14 (26.4%) studies, the entire sample–usually made up of formal sector workers or students–had formal education.

There was scant information on the lifestyle risk profile of the study participants. In 38 (44.7%) studies with available information, the prevalence of smoking was 0%-59.5% with a median of 3.4%. The prevalence of current alcohol consumption was 9.5%-37.6% in 20 studies and 41.1%-73.8% in 17 studies with available information. The prevalence of obesity ranged from 2.0% to 64% with a median of 14.1% in 56 (65.9%) studies with available information. The prevalence of overweight ranged from 6.2% to 44.0% with a median of 28.2% in 54 (63.5%) studies while that of overweight or obesity ranged from 0.8% to 84.0% with a median of 44.0% in 55 (64.7%) studies with available information.

Information on co-morbidities was even more limited. Twenty-one (24.7%) studies reported a prevalence of diabetes mellitus ranging from 1.2% to 25.2% with a median of 4.5%. Ten (11.8%) studies reported prevalence of impaired fasting glycaemia ranging from 1.7% to 31.4%, with a median of 10.9%. in 13 (15.3%) studies, the prevalence of hypercholesterolaemia ranged from 0.2% to 49.1% with a median of 14.8%. The prevalence of metabolic syndrome reported by three (3.5%) studies ranged from 7.4% to 18.0% with a median of 8.1%. The results on risk factors and co-morbidities in the primary studies were typically not presented in a form that will allow sub-group analysis in relation to hypertension.

### Blood pressure measurement

As with the demographic data, there was limited reporting of the protocol for blood pressure measurement. There was wide diversity in the BP measurement protocols in terms of frequency and interval of BP measurements, the body posture, part of the body on which the BP was taken and the type of BP device used. Except for two studies, all the studies measured the BP at a single visit (Table 2). Only one study conformed to international practice guidelines [124] by evaluating BP three weeks after initial visit in those who had never been diagnosed with hypertension and whose systolic blood pressure (SBP) >140 mmHg or diastolic blood pressure (DBP) >90 mmHg [59]. The other study involving multiple visits measured the BP of all study participants once daily over three days, during which period the investigator administered different modules of a health questionnaire and a series of 24-hour dietary recalls [108].

**Table 2:** Blood pressure measurement protocols.

Characteristic	Frequency	Percent
Number of visits		
Single	82	97.6
Multiple	2	2.4
Frequency of BP readings per visit		
One	2	2.9
Two	31	44.9
Three	34	49.3
Four	2	2.9
Initial rest time before BP taken		
5 minutes	28	57.1
10 minutes	15	30.6
15 minutes	3	6.1
>15 minutes	3	6.1
BP readings used in the analysis of hypertension		
Mean of 2 readings	40	59.7
Mean of last 2 of 3 readings	10	14.9
Mean of 3 readings	16	23.9
Other	1	1.5
Body posture for BP measurement		
Seated upright	67	97.1
Mixed	2	2.9
Body part on which BP taken		
right arm	18	36.7
left arm	23	46.9
any arm	5	10.2
each arm	1	2.0
wrist	2	4.1
Type of BP device		
electronic	54	69.2
manual mercury	15	19.2
manual aneroid	4	5.1
manual unspecified type	4	5.1
other	1	1.3
Location where BP taken		
field—home/workplace	76	97.4
health centre	2	2.6
public facility—school	1	1.3

In 69 studies with available information, 65 (94.2%) reported reading the BP twice (44.9%) or thrice (49.3%) at a visit. In the other studies, the BP was measured once or four times. Most of the BP readings were taken at five minutes interval or less. In some studies with longer intervals between measurements, the BP was taken at the start, middle and end of an interview process [78, 79]. In 67 (78.7%) studies with available information, the mean of two BP readings (59.7%), the last two of three BP readings (14.9%) or of all three BP readings (23.9%) were used in the analysis.

The BP was measured mostly in the seated position in 67 (97.1%) out of the 69 studies reporting on the body posture for the BP measurement. Out of 49 studies with available information, it was taken on the left arm in 23 (46.9%) studies or on the right arm in 18 (36.7%) studies. BP was measured in the lying position in one study [95] and in both the lying and upright positions in another study [81]. It was also taken on any arm [45, 73, 95, 111, 123], each arm [116] or on the wrist [49, 50].

In 78 (91.8%) studies, the electronic BP monitor was the most frequently used device in taking measurements (69.2%). In 23 (29.5%) studies, a manual sphygmomanometer (mercury– 15 studies, aneroid– 4 studies, unspecified type– 5 studies) was used while in one study [66] both the digital and manual devices were used. Blood pressures were measured at the home or workplace of the participants in 76 (96.2%) studies. In three (3.8%) studies, they were taken at a health centre or other public space [51, 95, 102].

Strategies to improve the quality of the BP measurements in various studies included the use of the same set of trained personnel, taking the readings between 7.00am and 10.00am, supporting the arm at the level of the heart and ensuring that subject’s feet were kept flat on floor, taking a third reading if the preceding two differed by >10 mmHg, changing the device’s battery after every 50 subjects, and using the appropriate cuff sizes.

### Prevalence of adult hypertension

Hypertension was defined based on BP ≥ 140/90 mmHg in 66 (77.6%) studies. It was based on diastolic hypertension in six (7.1%) studies [45, 55, 63, 88, 93, 114]; systolic hypertension in seven (8.2%) studies [78, 89, 108, 109, 115, 120, 121]; and on both systolic and diastolic hypertension in two (2.4%) studies [83, 112]. One (1.2%) study defined hypertension based on isolated systolic hypertension [92] and three studies (3.5%) on BP > 140/90 mmHg [84, 96, 123].

In 17 (20.0%) studies, the prevalence of hypertension was based on the measured BP≥ 140/90 mmHg as well as on or a history of previous diagnosis of hypertension by a health professional [49, 50, 72, 76, 80, 117] or on a history of current anti-hypertensive medication [43, 59, 62, 68, 71–74, 87, 91, 94, 98]. Six (7.1%) studies, five of them conducted by the same research team, excluded participants with a history of hypertension, diabetes, coronary heart disease or those on anti-hypertensive or cholesterol-lowering medication use [54, 65, 67, 69, 109, 125]. These studies involved biochemical tests on cardio-metabolic risk factors.

The blood pressure was read in 82,045 subjects, representing 97.5% of the total number enrolled. In individual studies, the prevalence of hypertension ranged from 2.8% among tertiary level students [90] to 67.5% among pastors and church workers [71]. In the study with the highest prevalence, 38% of the participants had previously been diagnosed with hypertension. In 51 and 50 individual studies respectively, the prevalence ranged from 2.8% [90] to 58.3% [72] in men and from 1.0% [122] to 90.0% [60] in women.

In 15 (17.6%) studies with available information, 42.7%-88.1% of those with hypertension had Grade 1 or mild hypertension with BP 140-159/90-99 mmHg while 11.9%-82.5% had Grade 2 or moderate or severe hypertension with BP ≥160/100 mmHg. In 11 (12.9%) studies, more than one-third of the subjects with hypertension had Grade 2 hypertension. In four of the studies reporting Grade 2 hypertension, 14.7%-19.2% of subjects had BP >180/110 mmHg (severe hypertension) [59, 74, 108, 116].

From the meta-analysis, the pooled prevalence of hypertension across the 85 studies was 27.0% [95% confidence interval (CI): 24.0%-30.0%]. In the studies providing sex-specific estimates, the pooled prevalence, from separate analysis, was similar in men (26.0%, 95% CI: 22.0%-30.0%) and in women (24%, 95% CI: 20.0%-27.0%) (S1 and S2 Figs). The age distribution of the men and women in each series was broadly similar, with 76.4% and 78.0% being younger than 50 years old. Information on the BMI status was scanty, being provided by 19 (37.3%) of the 51 studies in men and 19 (38.0%) of the 50 studies in women. A prevalence of obesity of 10% or higher was more frequently reported in the studies in women (17/19) than those in men (7/19).

The pooled prevalence of hypertension was significantly higher in studies which defined hypertension based on measured BP and history of previous diagnosis or current treatment with anti-hypertensive (34%, 95% CI: 31.0%-38.0%) than those based on measured BP alone (25.0%, 95% CI: 22.0%-29.0%) (S3 Fig).

**Fig 3:**
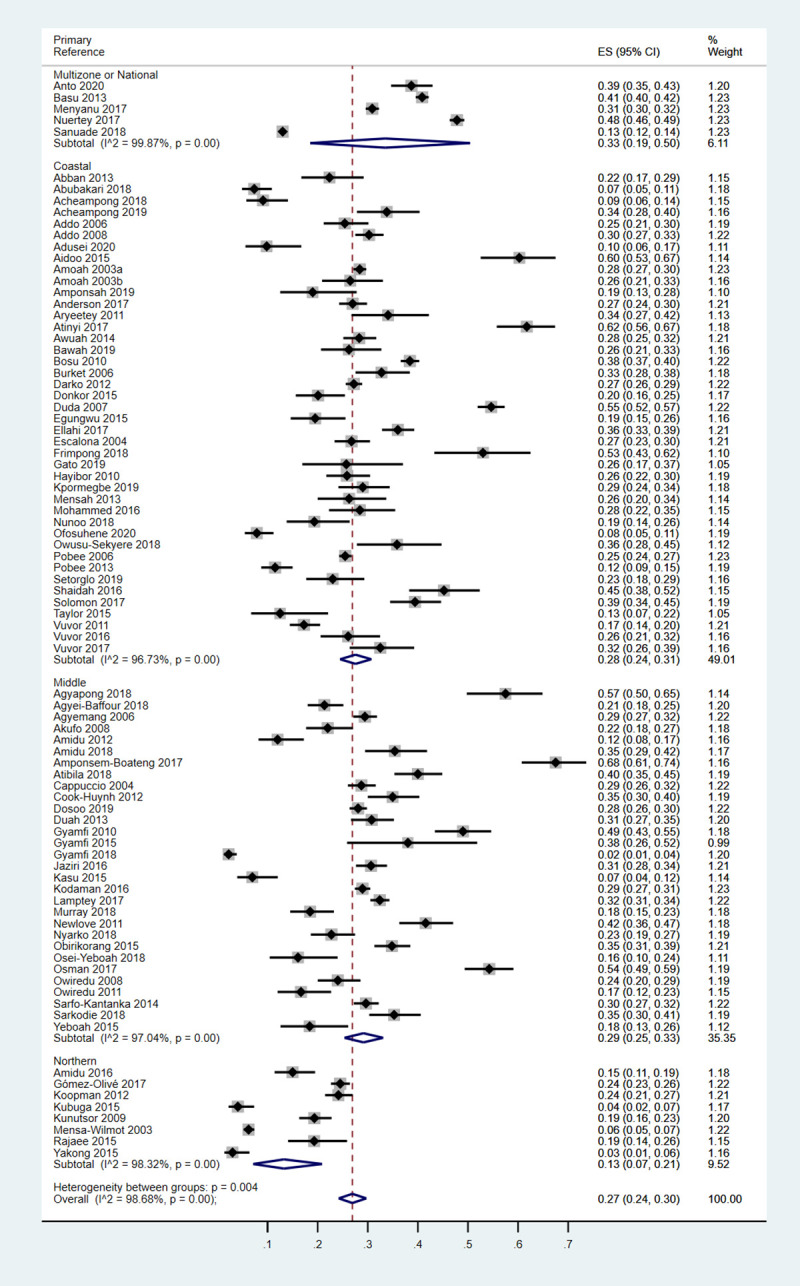
Forest plot of the prevalence of hypertension by geo-ecological belt of Ghana.

The pooled prevalence in the coastal (28.0%, 95% CI: 24.0%-31.0%) and middle (29.0%, 95% CI: 25.0%-33.0%) geo-ecological belts was twice that of the northern geo-ecological belt (13.0%, 95% CI: 7.0%-21.0%) (Fig 3). At the level of the administrative regions, the Volta Region had the highest pooled prevalence (39.0%, 95% CI: 29.0%-49.0%) while the Northern Region had the lowest prevalence (7.0%, 95% CI: 1.0%-15.0%).

The pooled prevalence of hypertension did not differ statistically by type of publication [original articles/abstracts (27.0%, 95% CI 24.0%-30.0%), dissertations (25.0%, 95% CI 18.0%-34.0%), other (39%, 95% CI 37.0%-40.0%)]; peer review status of publications [not peer reviewed (26.0%, 95% CI 18.0%-34.0%), peer reviewed (27.0%, 95% CI 24.0%-30.0%)]; multiplicity of publications [single publication (26.0%, 95% CI 23.0%-30.0%), multiple publications (29.0%, 95% CI 23.0%-35.0%)]; frequency of BP readings per visit [one (27.0%, 95% CI 24.0%-30.0%), two (25.0%, 95% CI 21.0%-29.0%), three (27.0%, 95% CI 22.0%-32.0%), four (28.0%, 95% CI 27.0%-30.0%)]; threshold for the definition of hypertension [BP ≥ 140/90 mmHg (27%, 95% CI 23.0%-30.0%), other subset definitions like systolic or diastolic hypertension (28.0%, 95% CI 23.0%-34.0%)]; sample size [<300 subjects (26.0%, 95% CI 21.0%-31.0%), 301–1000 subjects (27.0%, 95% CI 22.0%-31.0%), >1000 subjects (30.0%, 95% CI 24.0%-36.0%)]; age of participants [median age <50 years (25%, 95% CI 22.0%-28.0%), median age ≥50 years (36%, 95% CI 27.0%-44.0%)]; or urban-rural residence [rural residents (25.0%, 95% CI 18.0%-32.0%), urban residents (26.0%, 95% CI 23.0%-29.0%), mixed population (32.0%, 95% CI 26.0%-39.0%)] (S4–S6 Figs).

### Sources of heterogeneity

We found substantial heterogeneity among the included studies on the prevalence of hypertension [χ^2^_(84 df)_ = 6368.3; I^2^ = 98.7; p<0.001]. There was similarly high heterogeneity (I^2^ values ≥96.4%; p<0.001) in the subgroup analyses by type of publication, peer-reviewed status of publication, multiplicity of publications, definition of hypertension, sample size, age of participants, and type of residence. The chi-squared statistical tests for sub-group differences were statistically significant.

### Meta-regression analysis

We explored the study year, year of publication of the study, the sample screened for hypertension, percentage of obesity in the participants and the type of BP measuring device as potential sources of heterogeneity in univariate and multivariate meta-regression analyses. Only the percentage obesity was statistically significant, explaining 11.7% of the between-study heterogeneity in the univariate model (Table 3). However, in the combined model adjusted for the other covariates, the percentage heterogeneity explained declined slightly to 9.7% (I^2^ = 89.2, tau^2^ = 0.016).

**Table 3:** Meta-regression analysis of selected variables to explore potential sources of heterogeneity.

Model	N	Coefficient	95% CI	Tau^2^	Adj. R^2^ (%)	I^2^ (%)	p value
No covariates	85	0.282	0.252, 0.311	0.015		92.65	<0.001
Univariate							
Study year	81	8.45e-5	-0.004, 0.005	0.015	-1.77	93.01	0.97
Publication year	85	0.001	-0.006, 0.007	0.015	-1.54	92.71	0.839
Sample screened for hypertension	85	-1.10e-6	-1.61e-5, 1.39e-5	0.015	-1.73	91.25	0.884
Percentage obesity	56	0.004	-9.71e-4, 0.007	0.015	11.73	89.92	**0.012**
Device (ref: digital type)	85	-3.25e-4	1.41e-3, 7.58e-4	0.015	-1.02	92.74	0.553
Multiple covariates	53			0.016	9.67	89.24	0.1173
Study year	53	-6.65e-3	-0.028, 0.015				0.531
Publication year	53	0.007	-0.016, 0.031				0.535
Sample screened for hypertension	53	7.51e-6	-3.27e-5, 4.77e-5				0.709
Percentage obesity	53	0.005	1.54e-3, 0.009				**0.006**
Device type	53	-8.45e-4	-2.49e-3, 7.95e-4				0.305

CI = confidence interval

**Fig 4:**
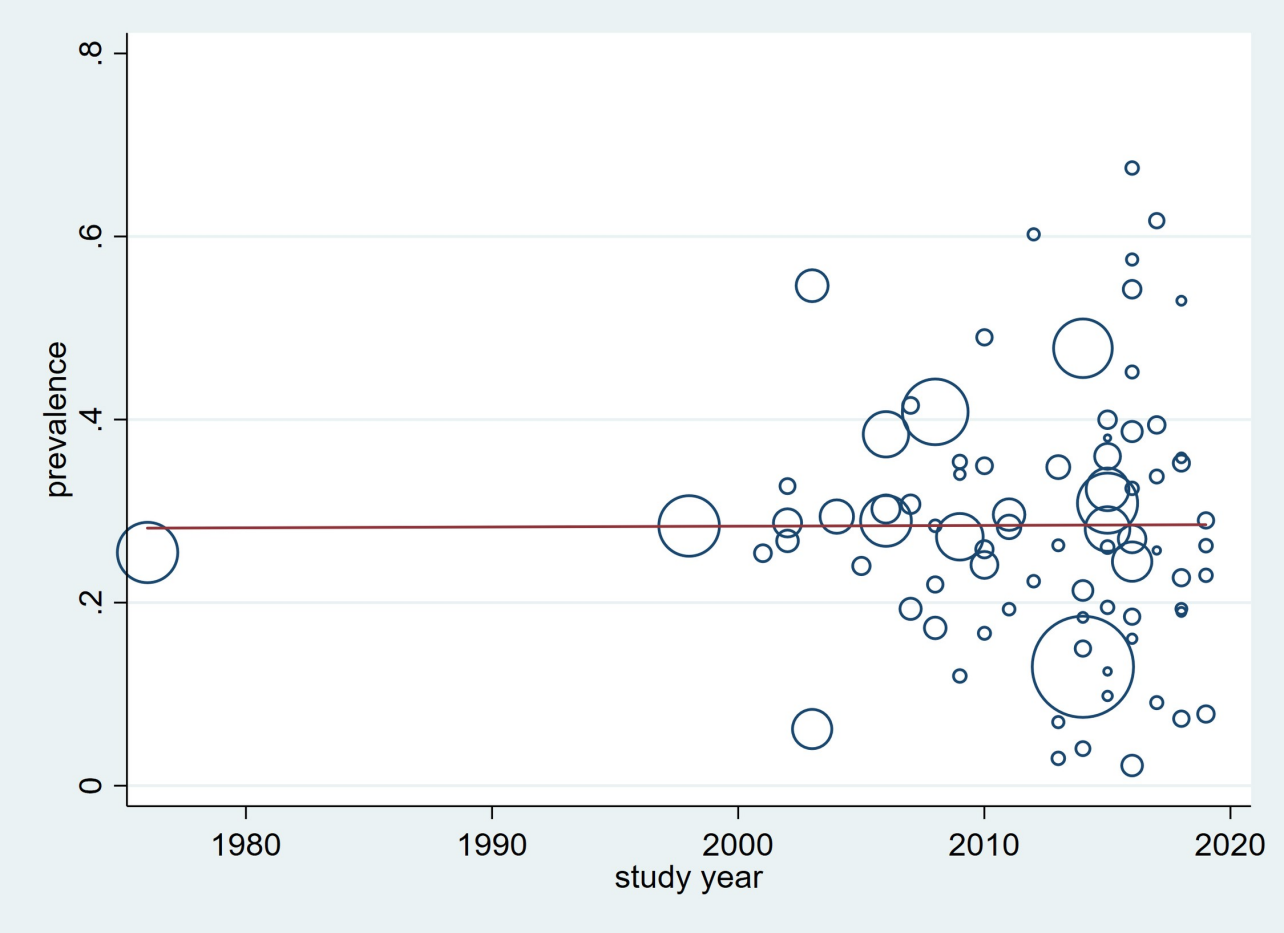
Trend in the prevalence of hypertension over study years 1976–2019.

The prevalence of hypertension did not appear to have changed over time, represented by the study year (Fig 4) or publication year (S7 Fig). There was no statistically significant variation in the prevalence over four decades of studies conducted since 1976 (p = 0.873). Neither was there any significant variation over the period of the publication year 2003–2020 (p = 0.839). There was still no clear trend in the prevalence of hypertension when observations were restricted to studies or publications in the past two decades.

### Evaluation of risk of bias and sensitivity analysis

Forty-nine (57.7%) studies were judged to be of high quality with low risk of bias while 15 (17.7%) studies were at moderate risk of bias (S4 Table). However, 20 (23.5%) studies were considered to be at high risk of bias. One study did not have sufficient information for its quality to be assessed [96]. The high-risk bias studies typically used convenient samples or volunteers. There was no association between the risk of bias and the peer-reviewed status of included studies (p = 0.619). The pooled prevalence of hypertension was similar for the different risk of bias ratings [low risk of bias (27.0%, 95% CI 24.0%-30.0%), moderate risk of bias (26.0%, 95% CI 18.0%-34.0%), high risk of bias (29.0%, 95% CI 24.0%-35.0%)].

**Fig 5:**
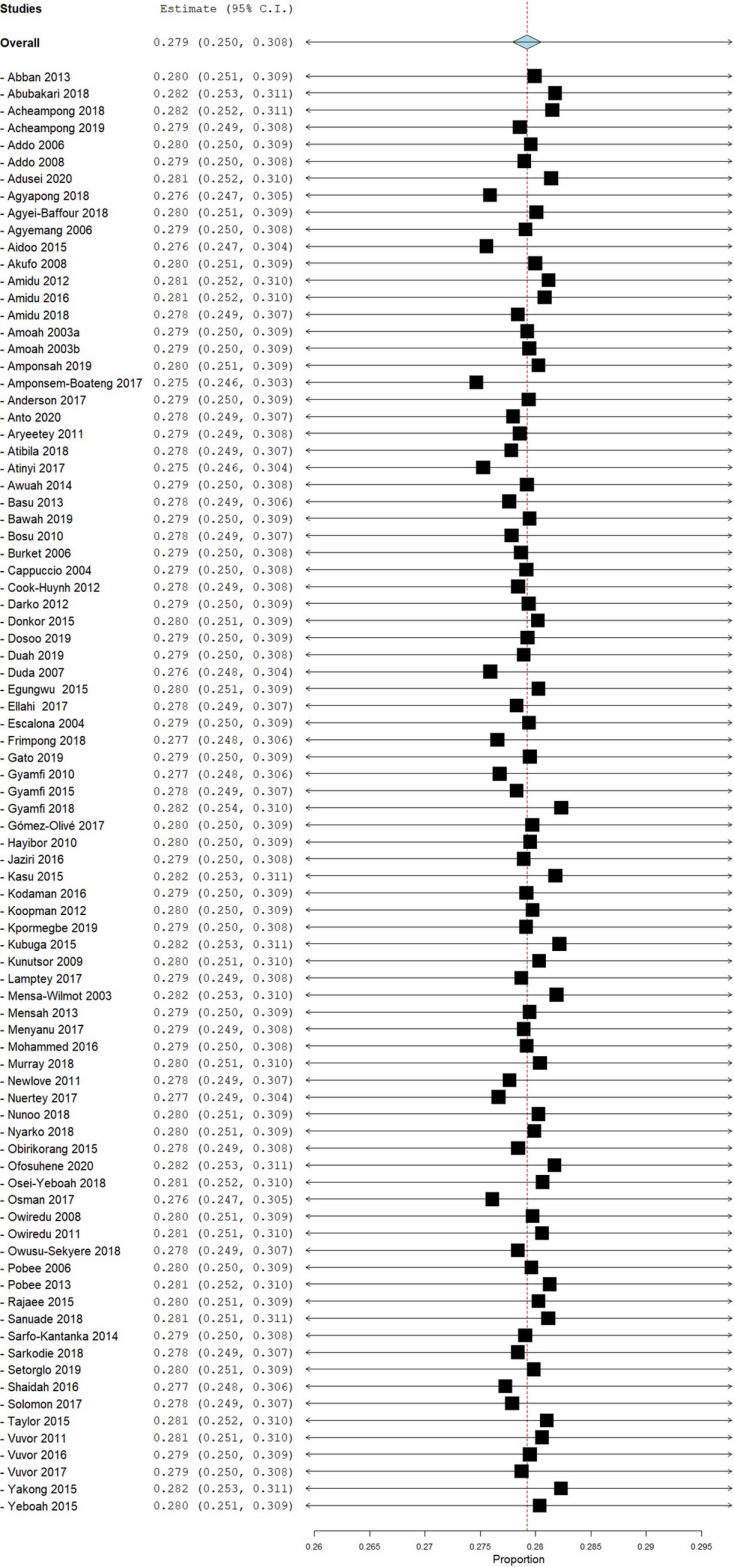
Leave-one-out sensitivity plot on studies on prevalence of hypertension in Ghana.

Eliminating all the high-risk bias studies from the analyses yielded a pooled prevalence of 27.0% (95% CI 24.%-30.0%) that was similar to the prevalence in the full set of studies. In the leave-one-out sensitivity analysis, no individual study was found to have any major impact on the pooled prevalence of hypertension (Fig 5). The omission of each study resulted in pooled prevalence that was within 0.4 percentage points of the original pooled estimate.

### Publication bias

**Fig 6:**
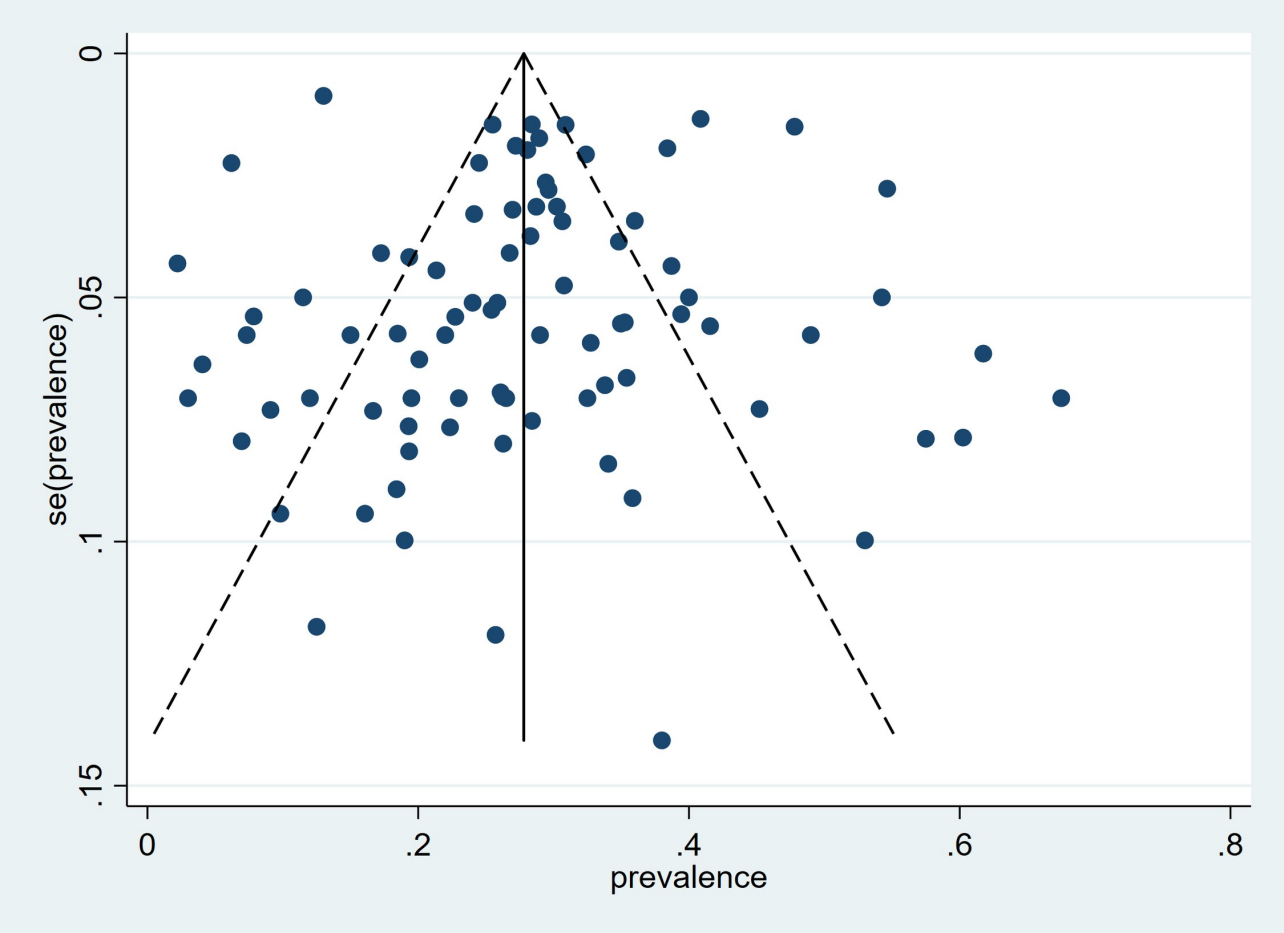
Funnel plot of included studies on the prevalence of hypertension in Ghana.

A funnel plot analysis of the included studies on the prevalence of hypertension did not show evidence of publication bias, as indicated by the plot symmetry (Fig 6). The Egger’s test was not statistically significant (p = 0.544).

### Prevalence of awareness, treatment and control

**Fig 7:**
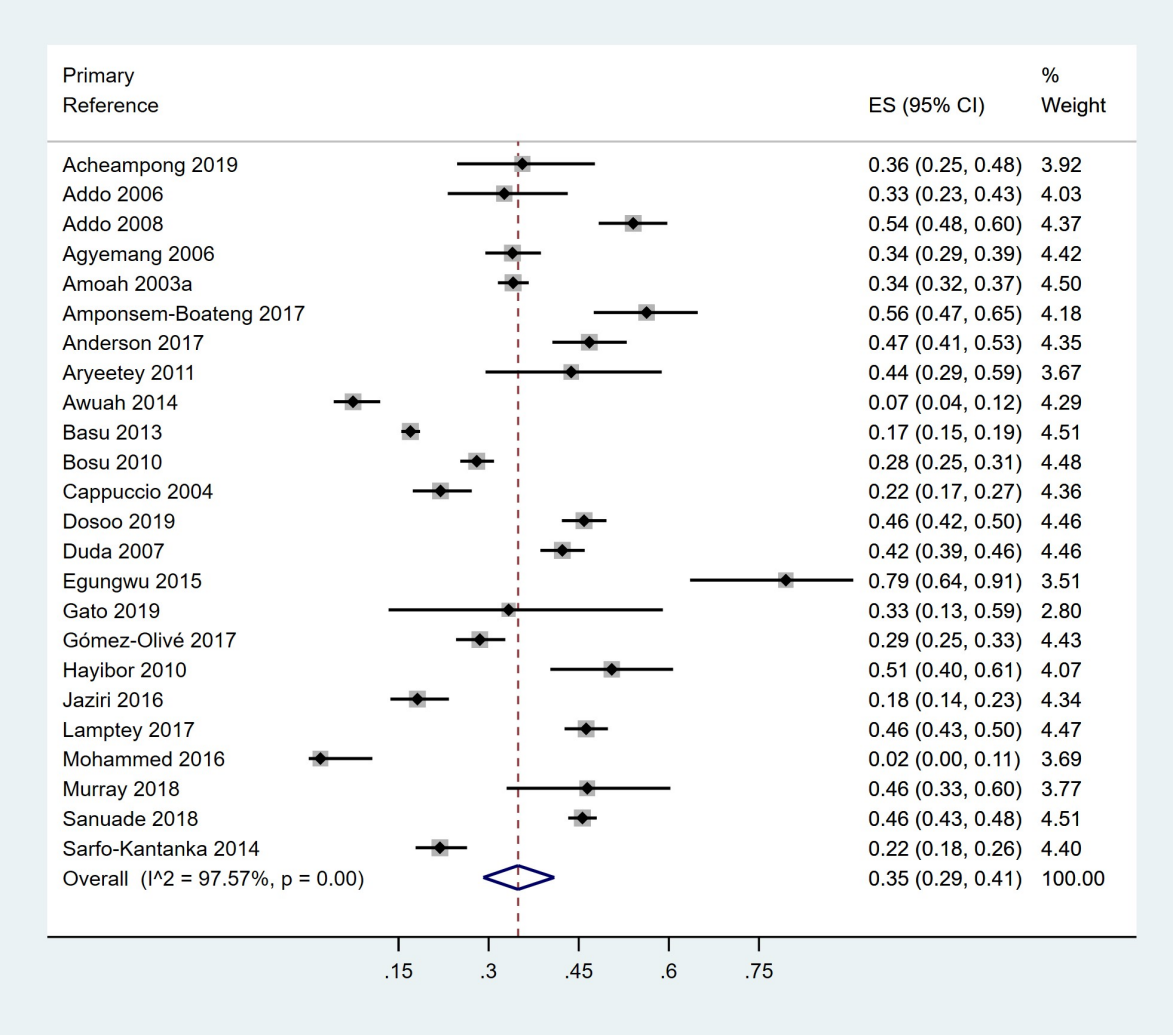
Forest plot of prevalence of awareness of hypertension in Ghana.

The pooled proportion of people with hypertension who were aware from a previous diagnosis in 24 studies was 35.0% (95% CI: 28.0%-40.0%) (Fig 7). Thus, nearly two-thirds of hypertension in Ghana was undiagnosed. In individual studies, the prevalence of awareness ranged from 2.0% among mostly poor residents in two peri-urban communities [101] to 79.5% among nurses at a regional hospital [82].

The pooled prevalence of subjects with hypertension who were currently on treatment with anti-hypertensive medication, from 17 studies, was 22.0% (95% CI: 16.0%-29.0%) (S8 Fig). In individual studies, the self-reported treatment ranged from 4.1% [98] to 46.4% [102]. Among those aware of their hypertensive status, 66.0% (95% C1: 52.0%-79.0%) were currently on anti-hypertensive treatment. An estimated 6.0% (95% CI: 3.0%-10.0%) of subjects with hypertension, from 16 studies, had their BP controlled. In individual studies, the proportion whose BP was controlled ranged from 1.0% [98] to 24.0% [52] (S9 Fig).

As with the prevalence of hypertension, the funnel plots for the prevalence of awareness, treatment and control did not show any evidence of publication bias (S10–S12 Figs). The Egger test p values were 0.440, 0.618 and 0.790 respectively.

## Discussion

To our knowledge, our study is the first in Ghana to publish an estimate of the prevalence of hypertension, awareness, treatment and control through a meta-analysis. There are several important findings that emerge from our analysis. We obtained a large number of studies from our search which met our inclusion criteria– 85 studies compared with 17 studies in our previous systematic review [12]. Thirteen studies from the earlier review were included in the current review. The rapid increase in the number of studies, particularly in the past decade, is likely due to several factors such as increase in the number of tertiary-level programmes in public health, dietetics, biochemistry, occupational hygiene; access to publicly available data from the DHS and SAGE; collaboration with external institutions and the greater availability of digital BP monitors which facilitates use by non-health professionals.

We observed a greater regional coverage of the studies with nine of the ten regions represented in the region-based studies in this review compared with four regions in our earlier review in 2010 [12]. Besides, there were five studies that were national in coverage in the current coverage whereas there was none in the earlier review. It is to be noted that, through the division of five existing regions in June 2019, Ghana’s ten administrative regions was increased to 16, of which 13 were represented in the subnational studies in this review.

With the greater regional spread of the included studies, the Greater Accra Region (GAR) and the Ashanti Region (AR), the two regions hosting the two largest cities in Ghana, were less represented in the region-based studies in the current review (52/79, 65.8%) than in our earlier review (14/17, 82.4%). However, there were fewer studies in northern belt than in the other geographical belts, perhaps a reflection of factors such as the distribution of health services, distance from capital, tertiary education, interest of health researchers and general socio-economic development in the country. To illustrate the potential contribution of tertiary programmes to hypertension research in Ghana, we note that, the University of Health Allied Sciences in Ho, which started operations in 2012, contributed four (66.7%) of the six publications on the hypertension in the Volta Region where it is located [45, 72, 83, 117]. A recent meta-analysis to estimate the prevalence of diabetes in Ghana could only obtain estimates for the northern belt based on self-reported diabetes in the nationwide SAGE study included in the 12 studies analyzed [11]. As the northern belt constitute a large part of the land mass of Ghana, it is essential to investigate further its relative low research output.

High as it is, we suspect our estimated prevalence (27.0%) of adult hypertension in Ghana is conservative. The use of restrictive definitions of hypertension; exclusion of participants previously diagnosed of hypertension as well as the assessment of hypertension from only BP measurements likely underestimated the prevalence of hypertension. As we found, the difference in the pooled prevalence of hypertension in studies using an expanded definition of hypertension including a history of previous diagnosis or antihypertensive treatment higher prevalence and that in those using definition based only on BP measurement was statistically significant.

Moreover, in multiple publications on the same study, we analyzed only the study that covered the entire sample rather than age-, sex or residential-subgroups [126–129] that could have inflated the prevalence. For example, we found upon enquiry from the researchers that three publications from the same authors on a rural community, Barekese, that we had thought came from a single study actually came from three different studies conducted in 2010–2012. The first of these studies in 425 adults aged ≥35 years reported a prevalence of hypertension of 44.7% [130]. Concerned about this high prevalence in the rural community, the investigators repeated the study the following year in 438 adults aged ≥35 years and confirmed their earlier study with an even higher prevalence of 50.9% [131]. Then, in 2012, the investigators conducted another study in the same community, this time sampling 845 adults aged ≥18 years and found a prevalence of isolated systolic hypertension of 30.6%. We chose to include only the last study in the meta-analysis because of its wider coverage although it reported the lowest prevalence. Besides, we expected that the prevalence of hypertension in this relatively small rural community will be stable over a short period of three years.

Ghana’s pooled prevalence of hypertension of 27.0% is similar to that of Nigeria (28.9%) [132], Cameroon (30.9%) [133] and SSA (30.0%-31.1%) [134, 135] but greater than that of Ethiopia (19.6%) [136] or of 44 developing countries (17.5%) [137]. The United Nations estimates that Ghana’s adult population in 2020 is about 19.54 million representing 62.9% of the total population. Applying the 27.0% prevalence to this figure translates into 5.27 million people who have hypertension. Somehow, this large number has been ‘invisible’ in the public health space, not only in Ghana but also in the global public health agenda [138, 139]. In contrast, HIV/AIDS which affects an estimated 340,000 persons in Ghana, receives more public health priority and is managed by a Commission under the Office of the President [140].

The apparent neglect of NCDs by several actors including policy makers, major multilateral and bilateral aid donors, and academics has long-been recognized [141]. In Ghana and elsewhere, it is evidenced by low funding, insufficient political commitment and persistent disease burden [142]. Between 2012 and 2014, the average global health development assistance provided for every disability-adjusted life year (DALY) associated with HIV/AIDS was US$30.84 compared with US$0.08 for NCDs [143]. The neglect of the control of hypertension is thought to be a driving factor for its persistence and the growing burden of stroke in Sub-Saharan Africa [144]. Leading authorities have decried the failure of the global community to translate the public health knowledge on the preventable causes of hypertension and the current clinical knowledge into effective prevention, treatment and control programmes [141, 145].

Based on an estimated prevalence of hypertension (BP ≥160/95 mmHg) of 5% in 1979, Pobee and colleagues warned of a silent epidemic of hypertension in Ghana, viewed against the assertion 50 years earlier that hypertension was rare in Africans [146]. Inspired by this observation, we described an ‘epidemic of hypertension’ in our systematic review of 2010 [12]. Since then, a rural epidemic of hypertension [131] and a national epidemic of obesity in Ghana have been described [18]. Of particular concern is our finding that the urban-rural gap in the prevalence of hypertension has become blurred and that the high prevalence has persistent over four decades. Moreover, up to a fifth of those with hypertension may have it as a severe grade indicating a long duration of poor control. Nearly two-thirds of adults with hypertension in Ghana are unaware of their status and so will be unable to initiate measures to control it. This estimate of undiagnosed hypertension is better than the 73% estimated for SSA but that for the poor control of hypertension in the two settings is similar [134].

There are misconceptions and poor knowledge of hypertension in Ghana even among patients living with hypertension [61, 147]. As in other countries, the population infrequently go for medical check-ups, preferring to go to the clinic only when they have symptomatic illness [29]. The prevalence of undiagnosed hypertension is relatively high even among health workers [43]. The national health insurance scheme and the organization of mass screening campaigns in public spaces such as places of worship, health facilities, pharmacies, recreational parks, shopping centres, marketplaces, universities, workplaces, and community centres present an opportunity for the population to know their BPs [148, 149]. Health education has been shown to be beneficial in improving knowledge of hypertension and healthier self-care practices [150]. Health workers in Ghana have identified barriers relating to health professionals, health system and patients which hinder adequate control of hypertension [151]. Some of these barriers could be addressed through better community engagement and the implementation of nurse-led interventions in task-shifting programmes [152, 153].

The combination of the enabling factors of high prevalence, low awareness and poor control of hypertension calls for an urgent response in line with Ghana’s strategies to prioritize cardiovascular health through investments in risk reduction dietary and lifestyle behaviour [154, 155]. In a recent evaluation, local experts in Ghana gave a low rating to the implementation of three-quarters of 43 indicators of health food environment [156]. Other experts cite inadequate resources and a focus on clinical rather than preventive actions as the major challenges hindering the implementation of diabetes and hypertension policies in Ghana [157].

Data on national trends of hypertension in Africa are scanty and present mixed results. For example, Mozambique reported a statistically significant increase from 33.1% to 38.9% between 2004 and 2015 from two national surveys [158] while Seychelles reported a stable age-standardized prevalence of 40% in surveys in 1989 and 2004 [159]. In Nigeria, the pooled prevalence among adults aged ≥20 years was projected to increase from 28.0% (95% CI 24.6%-31.9%) in 2010 to 30.8% (95% CI: 24.5%-33.7%) in 2030 [132]. Available studies in Africa generally project a stable [160, 161] or upward trend [30, 162] in adult hypertension at different periods between 1975 and 2030.

Our finding of similar pooled prevalence of hypertension in men and women in Ghana is consistent with that from other studies in Ghana, Cameroon and Nigeria [12, 132, 133]. However, in comparisons of sex differences in primary studies on the prevalence of hypertension which control for various confounders, male sex has been a key determinant among workers in West Africa [163] while female sex has been a key determinant among older adults in Africa [9].

Our meta-analysis showed a statistically significant lower prevalence of hypertension in the northern geo-ecological belt of Ghana than in the middle or coastal belts. The pattern is confirmed in individual nationwide studies where the prevalence in the northern regions is about a quarter to half that in the GAR [52, 127]. The lower prevalence has been attributed to traditional lifestyles in the northern belt characterized by manual farming and housekeeping and to the greater mobility of most people on foot or by bicycle [95, 97]. In one study, about 90% of the participants reported being physically active [100] and in another only 0.8% of the participants lived with overweight or obesity [95]. In demographic and health surveys, tobacco use in men and women in the northern belt has consistently been highest in Ghana but its influence on hypertension appears to be offset by the lean body-build, physical activity and traditional diets of the population [164]. A similar north-south divide is seen in nearby Benin where the prevalence in the northern regions of Atakora and Alibori is about one-third that in the Littoral region in which the capital city is located [165]. Health authorities are encouraged to work with key actors to maintain or reduce the low prevalence in the northern belt.

Until now, the GAR and AR have been considered the regions with high prevalence of hypertension in Ghana [52, 127]. Our findings show that regions such as the Volta, Eastern, Bono and Western have similar or higher prevalence of hypertension. In both rural and urban settings, the prevalence of hypertension was similar. The narrowing urban-rural gap in the prevalence of hypertension may be due to changing lifestyles with a lower physical activity and a shift from traditional diets rich in fibre to energy-dense processed foods high in fat, sugar and salt [40, 166]. With up to half of adults in some rural communities in Ghana living with hypertension [131] and about one-quarter living with overweight or obesity [18], cardiovascular risk should be now considered more pervasive in Ghana and resource planning and allocation adjusted accordingly.

### Strengths and limitations

Our study’s strengths are that it presents the first published pooled estimate of the prevalence of hypertension, awareness, treatment and control derived from studies more than four decades of studies covering the entire country; it employed a comprehensive search strategy across several data sources including unpublished data; it involved a large number of studies and study participants; and conforms to the standards of PRISMA reporting [23]. Our estimates are robust and corroborated by sensitivity analyses and the absence of evidence of a publication bias.

Although not available annually, the prevalence of hypertension is one of the sector wide indicators of Ghana uses to assess its annual health sector performance [167]. Like other African countries, Ghana faces the challenge of how to effectively monitor the size and trends of the hypertension epidemic in the country from reliable and accurate data sources. Ghana undertook a subnational STEPS survey of risk factors for chronic NCDs in 2006 and has been seeking to conduct a second (national) survey. However, in 2014, Ghana became one of six African countries to include BP assessment in its five-yearly national GDHS surveys [10, 168]. Having successfully conducted six rounds of GDHS surveys since 1988, there is good prospect to obtain periodic national estimates of the prevalence of hypertension at lower costs than through separate stand-alone STEPS surveys. The much lower estimate of prevalence of hypertension in the GDHS 2014 of 13.0% which is half of the pooled estimate from the current meta-analysis could be misinterpreted as successful control of hypertension whereas it may reflect differences in the study population and in the methodology. While DHS has been slow to include the assessment of biomarkers, estimates on the prevalence of diabetes using fasting blood glucose or glycosylated haemoglobin for three countries (Bangladesh, Namibia, Uzbekistan) are now available [168].

Ghana could also exploit data from its demographic surveillance sites to monitor the incidence and prevalence of hypertension. Two of these sites (Kintampo, Navrongo) contributed studies to this meta-analysis [80, 87, 97, 100]. Ghana could also monitor hypertension from the three waves of SAGE studies that have been conducted three waves since 2008, two of which are included in the current meta-analysis [49, 50]. The prevalence in Ghana declined from 40.9% in the SAGE Wave 1 in 2008 to 30.9% in SAGE Wave 2 in 2014–15 but rose to 37.6% in Wave 3 in 2018–19 [49, 50, 169]. However, given the challenges in descriptively triangulating the possibly divergent prevalence data, our meta-analysis provides a useful tool to statistically synthesize these empirical data.

Nonetheless, our study has some limitations. First is the problem of missing information on variables such as the age- and sex-specific prevalence, participation rate, or the BP measurement protocol. Secondly, all but two of the studies assessed BP at a single visit. This has the effect of overestimating the true prevalence of the hypertension [170]. The scale of overestimation is shown by Addo et al where the prevalence of hypertension based on an initial screening was 37.5% but dropped to 30.2% after evaluation three weeks later [59]. Similarly, among a subset of study participants in rural northern Ghana, the mean SBP dropped from 125.7±22.6 mmHg to 121.5±19.6 mmHg and the mean DBP from 72.2±12.4 mmHg to 70.5±11.9 mmHg two weeks after the initial assessment [97]. Such anomalies explain why the International Society of Hypertension recommends that a diagnosis of hypertension be made after 2–3 office visits at 1–4-week intervals (depending on the BP level), except where the BP≥ 180/110 mmHg and there is evidence of cardiovascular disease [124].

Thirdly, there was substantial heterogeneity across the included studies which undermines confidence in the pooled estimate. As might be expected with the large number of studies, there were differences in the study population, study protocols and assessments. We excluded several variables such as age, type of publication, sample size as the potential sources of the heterogeneity. There was some evidence of percentage of obesity in the sample contributing to this heterogeneity. High levels of heterogeneity appear to be the bane of similar meta-analytic studies on hypertension in Africa with reports of I^2^ statistics of 98.2%-99.5% [132–136].

Lastly, we found a relatively high number of studies with potentially-biased estimates of the prevalence of hypertension. However, our sensitivity analyses showed that no single study had a major impact on our pooled estimate of hypertension. Neither did eliminating high-risk bias studies have any significant effect. Overall, we are confident about the robustness of our pooled estimates on the prevalence of hypertension in apparently-healthy persons living in Ghana.

## Conclusions

Our systematic review and meta-analysis covering a large number of studies and of study participants across the country suggests that, conservatively, more than one in four adults in Ghana has hypertension. This prevalence of hypertension appears to have persisted over a long time and has reached levels in rural populations that are similar to that in urban populations. Hypertension may be less common in the northern sector of the country than in other regions. The hypertension hotspots are no longer only the Greater Accra and Ashanti Regions but also several other regions in the coastal and middle geographic zones of the country. Most people in Ghana with hypertension are not aware they have it and its control is disturbingly low.

We call for further studies to better understand the drivers of the hypertension epidemic in Ghana. This notwithstanding, Ghana has sufficient information to prioritize the cardiovascular health of its people towards meeting the global target of a 25% reduction in the 2010 age-standardized prevalence of hypertension by the year 2025 [171]. Strong political commitment and concerted whole-of-government and whole-of-society actions are needed to implement best-buy interventions such as fiscal levers, salt reduction, health promotion, promotion of physical activity which have been successful in reducing both systolic and diastolic BP in Sub-Saharan Africa [172, 173].

## Supporting information

S1 Table Preferred Reporting Items for Systematic Reviews and Meta-Analysis (PRISMA) 2009 checklist. (DOC)

S2 Table Search strategy for Ovid Medline and Ovid Embase databases. (DOCX)

S3 Table Multiple publications (linked to their primary studies) excluded from the meta-analysis. (DOCX)

S4 Table Evaluation of risk of bias in included studies on systematic review of hypertension in Ghana. (DOCX)

S1 Fig Forest plot of the prevalence of hypertension in men. (TIF)

S2 Fig Forest plot of the prevalence of hypertension in women. (TIF)

S3 Fig Forest plot of the prevalence of hypertension by scope of its definition. (TIF)

S4 Fig Forest plot of the prevalence of hypertension by peer-reviewed status of publication. (TIF)

S5 Fig Forest plot of the prevalence of hypertension by median age of participants. (TIF)

S6 Fig Forest plot of the prevalence of hypertension by urban-rural residence. (TIF)

S7 Fig Trends in the prevalence of hypertension over publication year 2003–2020. (TIF)

S8 Fig Forest plot of the prevalence of current treatment among subjects with hypertension. (TIF)

S9 Fig Forest plot of the prevalence of controlled blood pressure among subjects with hypertension. (TIF)

S10 Fig Funnel plot of studies reporting awareness of hypertension. (TIF)

S11 Fig Funnel plot of studies reporting treatment of hypertension. (TIF)

S12 Fig Funnel plot of studies reporting control of hypertension. (TIF)
