# Supplementary material for: Prevalence, awareness and control of hypertension in Ghana: A systematic review and meta-analysis
Source: PLoS One. 2021 Mar 5;16(3):e0248137. doi: 10.1371/journal.pone.0248137 (PMC7935309; doi:10.1371/journal.pone.0248137)
Supplement: S2 Table — (DOCX) [file pone.0248137.s002.docx]

## S2 Table Search strategy in integrated PubMed and Embase on the prevalence of hypertension in Ghana

| **No.** | **Search terms** |
| --- | --- |
| 1 | exp hypertension/ep, et, pc [Epidemiology, Etiology, Prevention] |
| 2 | (Hypertensi* or "blood pressure" or cardiovascular or cardiometabolic).ab. |
| 4 | exp prevalence/ |
| 5 | \| exp incidence/ \| \| --- \| |
| 6 | (Prevalence or proportion or survey or descriptive or cross-sectional or cohort or longitudinal or "attributable fraction" or incidence).ab. |
| 7 | 4 or 5 or 6 |
| 8 | ghana*.sh. |
| 9 | 3 and 7 and 8 |
| 10 | remove duplicates from 9 |
| 11 | limit 10 to ("all adult (19 plus years)" or "adolescent (13 to 18 years)") [Limit not valid in Embase; records were retained] |
