# Supplementary material for: Prevalence, awareness and control of hypertension in Ghana: A systematic review and meta-analysis
Source: PLoS One. 2021 Mar 5;16(3):e0248137. doi: 10.1371/journal.pone.0248137 (PMC7935309; doi:10.1371/journal.pone.0248137)
Supplement: S3 Table — (DOCX) [file pone.0248137.s003.docx]

## S3 Table

## Multiple publications (linked to their primary studies) excluded from the meta-analysis

| **Serial #** | **Author (Year)** | **Title** | **Article type** | **Primary study** | **Primary study Ref #** |
| --- | --- | --- | --- | --- | --- |
| **1** | Addo 2001 | Hypertension in rural Ga- a study of four rural communities in Ghana | Dissertation | Addo 2006 | [32] |
| **2** | Addo 2009 | Socioeconomic position and hypertension: A study of urban civil servants in Ghana | Original article | Addo 2008 | [63] |
| **3** | Addo 2009 | Hypertensive target organ damage in Ghanaian civil servants with hypertension | Original article | Addo 2008 | [63] |
| **4** | Anonymous 2008 | High blood pressure in urban Ghana | Abstract | Addo 2008 | [63] |
| **5** | Adusei 2015 | Mapping of health conditions associated with e-waste activities at Agbogbloshie, Accra | Dissertation | Adusei 2020 | [33] |
| **6** | Agyemang 2006 | Factors associated with hypertension awareness, treatment, and control in Ghana, West Africa | Original article | Agyemang 2006 | [85] |
| **7** | Agyemang 2008 | Prehypertension in the Ashanti region of Ghana, West Africa: An opportunity for early prevention of clinical hypertension | Original article | Agyemang 2006 | [85] |
| **8** | Tandoh 2017 | State of hypertension, diabetes mellitus and anemia among residents of selected communities in Ghana | Original article | Anderson 2017 | [45] |
| **9** | Ansong 2009 | Dietary Habits, Obesity and Elevated Blood Pressure among Workers of the College of Health Sciences. | Dissertation | Aryeetey 2011 | [35] |
| **10** | Atibila 2015 | Assessment of risk factors for hypertension in Dormaa Municipality (Ghana) | Dissertation | Atibila 2018 | [34] |
| **11** | Afrifa-Anane 2015 | The association of physical activity, body mass index and the blood pressure levels among urban poor youth in Accra, Ghana | Original article | Awuah 2014 | [65] |
| **12** | Sowah 2013 | Lifestyle, perception of health and blood pressure among Accra's urban poor | Dissertation | Awuah 2014 | [65] |
| **13** | Agrawal 2016 | Association between body mass index and prevalence of multimorbidity in low-and middle-income countries: a cross-sectional study | Original article | Basu 2013 | [41] |
| **14** | Arokiasamy 2017 | Chronic noncommunicable diseases in 6 low- and middle-income countries: Findings from wave 1 of the world health organization's Study on Global Ageing and Adult Health (SAGE) | Original article | Basu 2013 | [41] |
| **15** | Boakye 2017 | Socioeconomic Disparities in the Prevalence of Cardiometabolic Risk Factors in Ghanaian Women | Original article | Basu 2013 | [41] |
| **16** | Boateng 2015 | Examining the Risk Factors Associated With Hypertension Among the Elderly in Ghana. | Original article | Basu 2013 | [41] |
| **17** | Boateng 2017 | Obesity and the burden of health risks among the elderly in Ghana: A population study. | Original article | Basu 2013 | [41] |
| **18** | Capistrant 2019 | Do determinants of hypertension status vary between Ghana and South Africa? Study on global ageing and adult health: hypertension in sub-Saharan Africa | Original article | Basu 2013 | [41] |
| **19** | Gebreselassie 2015 | Epidemiology of Hypertension Stages in Two Countries in Sub-Sahara Africa: Factors Associated with Hypertension Stages | Original article | Basu 2013 | [41] |
| **20** | Koyanagi 2014 | Chronic conditions and sleep problems among adults aged 50 years or over in nine countries: A multi-country study | Original article | Basu 2013 | [41] |
| **21** | Lloyd-Sherlock 2014 | Hypertension among older adults in low and middle-income countries: Prevalence, awareness and control | Original article | Basu 2013 | [41] |
| **22** | Lloyd-Sherlock 2017 | Diseases of the Rich? The Social Patterning of Hypertension in Six Low- and Middle-Income Countries. | Original article | Basu 2013 | [41] |
| **23** | Maurer 2015 | One-year routine opportunistic screening for hypertension in formal medical settings and potential improvements in hypertension awareness among older persons in developing countries: Evidence from the study on global ageing and adult health (SAGE) | Original article | Basu 2013 | [41] |
| **24** | Minicuci 2014 | Sociodemographic and socioeconomic patterns of chronic noncommunicable disease among the older adult population in Ghana | Original article | Basu 2013 | [41] |
| **25** | Sanuade 2019 | Prevalence and correlates of stroke among older adults in Ghana: Evidence from the Study on Global AGEing and adult health (SAGE) | Original article | Basu 2013 | [41] |
| **26** | Tenkorang 2015 | Validity of Self-Report Data in Hypertension Research: Findings From The Study on Global Ageing and Adult Health | Original article | Basu 2013 | [41] |
| **27** | Tyrovolas 2015 | Determinants of the components of arterial pressure among older adults - The role of anthropometric and clinical factors: A multi-continent study | Original article | Basu 2013 | [41] |
| **28** | Wu 2015 | Common risk factors for chronic non-communicable diseases among older adults in China, Ghana, Mexico, India, Russia and South Africa: the study on global AGEing and adult health (SAGE) wave 1 | Original article | Basu 2013 | [41] |
| **29** | Yang 2016 | Prevalence, awareness, treatment, and control of hypertension in the older population: results from the multiple national studies on ageing | Original article | Basu 2013 | [41] |
| **30** | Banerjee 2018 | Pulse Pressure Relationships with Demographics and Kidney Function in Ashanti, Ghana | Original article | Cappuccio 2004 | [52] |
| **31** | Cappuccio 2003 | Prevalence, detection, management and control of hypertension in Ashanti, West Africa. II. Gender differences | Abstract | Cappuccio 2004 | [52] |
| **32** | Cappuccio 2003 | Prevalence, detection, management and control of hypertension in Ashanti, West Africa. I. Differences between semi-urban and rural areas | Abstract | Cappuccio 2004 | [52] |
| **33** | Cappuccio 2004 | Prevalence, detection, management and control of hypertension in Ashanti, West Africa | Abstract | Cappuccio 2004 | [52] |
| **34** | Cappuccio 2006 | A community programme to reduce salt intake and blood pressure in Ghana ISRCTN88789643 | Original article | Cappuccio 2004 | [52] |
| **35** | Kerry 2005 | Blood pressure and body mass index in lean rural and semi-urban subjects in West Africa | Original article | Cappuccio 2004 | [52] |
| **36** | Werts 2012 | Prevalence of hypertension and associated risk factors in Adansi South, Ghana | Abstract | Duah 2013 | [142] |
| **37** | Duda 2011 | The Health of the "Older Women" in Accra, Ghana: Results of the Women's Health Study of Accra | Original article | Duda 2007 | [50] |
| **38** | Hill 2007 | Health of urban Ghanaian women as identified by the Women's Health Study of Accra | Original article | Duda 2007 | [50] |
| **39** | Duda 2007 | Prevalence of Obesity in Women of Accra, Ghana | Original article | Duda 2007 | [50] |
| **40** | George 2019 | Kidney damage and associated risk factors in rural and urban sub-Saharan Africa (AWI-Gen): a cross-sectional population study | Original article | Gomez-Olive 2017 | [91] |
| **41** | Williams 2013 | The burden and correlates of hypertension in rural Ghana: A cross-sectional study | Original article | Jaziri 2016 | [79] |
| **42** | Williams 2014 | Silent Crisis: Epidemic Hypertension in Rural West Africa | Original article | Jaziri 2016 | [79] |
| **43** | Koopman 2014 | Scarcity of atrial fibrillation in a traditional African population: A community-based study | Original article | Koopman 2012 | [64] |
| **44** | Kunutsor 2012 | Cardiovascular risk in a rural adult West African population: Is resting heart rate also relevant? | Abstract | Kunutsor 2009 | [125] |
| **45** | Kunutsor 2014 | Cardiovascular risk in a rural adult West African population: Is resting heart rate also relevant? | Original article | Kunutsor 2009 | [125] |
| **46** | Aheto 2019 | Multilevel modelling, prevalence and predictors of hypertension in Ghana: Evidence from Wave 2 of the World Health Organization’s Study on Global AGEing and adult health | Original article | Menyanu 2017 | [42] |
| **47** | Newlove 2012 | The relationship between lifestyle variables, blood pressure and dietary habits of male adult miners in Ghana. | Original article | Newlove 2011 | [57] |
| **48** | Amegah 2018 | Educational attainment modifies the association of wealth status with elevated blood pressure in the Ghanaian population | Original article | Sanuade 2018 | [44] |
| **49** | Amegah 2019 | Cooking with shea butter is associated with lower blood pressure in the Ghanaian population | Original article | Sanuade 2018 | [44] |
| **50** | Arku 2018 | Elevated blood pressure and household solid fuel use in premenopausal women: Analysis of 12 Demographic and Health Surveys (DHS) from 10 countries | Original article | Sanuade 2018 | [44] |
| **51** | Dzokoto 2019 | Risk factors for hypertension among Ghanaian: Evidence from Ghana demographic and health survey | Original article | Sanuade 2018 | [44] |
| **52** | Oyekale 2019 | Effect of obesity and other risk factors on hypertension among women of reproductive age in Ghana: An instrumental variable probit model | Original article | Sanuade 2018 | [44] |
| **53** | Tuoyire 2019 | Gender differences in the association between marital status and hypertension in Ghana | Original article | Sanuade 2018 | [44] |
| **54** | Yaya 2018 | Differentials in prevalence and correlates of metabolic risk factors of non-communicable diseases among women in sub-Saharan Africa: evidence from 33 countries | Original article | Sanuade 2018 | [44] |
| **55** | Vuvor 2013 | Population-based study of diabetes mellitus prevalence and its associated factors in adult Ghanaians in the greater accra region | Abstract | Vuvor 2011 | [155] |
