# Supplementary material for: Prevalence, awareness and control of hypertension in Ghana: A systematic review and meta-analysis
Source: PLoS One. 2021 Mar 5;16(3):e0248137. doi: 10.1371/journal.pone.0248137 (PMC7935309; doi:10.1371/journal.pone.0248137)
Supplement: S4 Table — (DOCX) [file pone.0248137.s004.docx]

## S4 Table. Evaluation of risk of bias in included studies on systematic review of hypertension in Ghana

| *Primary Reference* | *Was the study's target population a close representation of the national population in relation to relevant variables?* | *Was the sampling frame a true or close representation of the target population?* | *Was some form of random selection used to select the sample, OR, was a census undertaken?* | *Was the likelihood of non-response bias minimal?* | *Was analysis performed to show no significant difference in relevant demographic characteristics between responders and non-responders?* | *Were data collected directly from the subjects (as opposed to a proxy)?* | *Was an acceptable case definition used in the study?* | *Was the study instrument that measured the parameter of interest?* | *Was the same mode of data collection used for all subjects?* | *Was the length of the shortest prevalence period for the parameter of interest appropriate?* | *Were the numerator(s) and denominator(s) for the parameter of interest appropriate?* | ***Overall risk*** |
| --- | --- | --- | --- | --- | --- | --- | --- | --- | --- | --- | --- | --- |
| Abban 2013 | Low risk | Low risk | Low risk | Low risk | High risk | Low risk | Low risk | Low risk | Low risk | Low risk | Low risk | **Low risk** |
| Abubakari 2018 | Low risk | Low risk | Low risk | Low risk | High risk | Low risk | Low risk | Low risk | Low risk | Low risk | Low risk | **Low risk** |
| Acheampong 2018 | Low risk | Low risk | Low risk | Low risk | High risk | Low risk | Low risk | Low risk | Low risk | Low risk | Low risk | **Low risk** |
| Acheampong 2019 | Low risk | Low risk | Low risk | Low risk | High risk | Low risk | Low risk | Low risk | Low risk | Low risk | Low risk | **Low risk** |
| Addo 2006 | Low risk | Low risk | Low risk | Low risk | High risk | Low risk | Low risk | Low risk | Low risk | Low risk | Low risk | **Low risk** |
| Addo 2008 | Low risk | Low risk | Low risk | Low risk | Low risk | Low risk | Low risk | Low risk | Low risk | Low risk | Low risk | **Low risk** |
| Adusei 2020 | Low risk | Low risk | Low risk | Low risk | High risk | Low risk | Low risk | Low risk | Low risk | Low risk | Low risk | **Low risk** |
| Agyapong 2018 | Low risk | Low risk | Low risk | High risk | High risk | Low risk | Low risk | Low risk | Low risk | Low risk | Low risk | **Low risk** |
| Agyei-Baffour 2018 | Low risk | Low risk | Low risk | Low risk | High risk | Low risk | Low risk | Low risk | Low risk | Low risk | Low risk | **Low risk** |
| Agyemang 2006 | High risk | Low risk | Low risk | Low risk | High risk | Low risk | Low risk | Low risk | Low risk | Low risk | Low risk | **Moderate risk** |
| Aidoo 2015 | High risk | High risk | High risk | High risk | High risk | Low risk | Low risk | Low risk | Low risk | Low risk | Low risk | **High risk** |
| Akufo 2008 | Low risk | Low risk | Low risk | Low risk | High risk | Low risk | Low risk | Low risk | Low risk | Low risk | Low risk | **Low risk** |
| Amidu 2012 | High risk | High risk | High risk | Low risk | High risk | Low risk | Low risk | Low risk | Low risk | Low risk | Low risk | **High risk** |
| Amidu 2016 | Low risk | High risk | High risk | Low risk | High risk | Low risk | Low risk | Low risk | Low risk | Low risk | Low risk | **Moderate risk** |
| Amidu 2018 | High risk | High risk | High risk | High risk | High risk | Low risk | Low risk | Low risk | Low risk | Low risk | Low risk | **High risk** |
| Amoah 2003a | Low risk | Low risk | Low risk | Low risk | Low risk | Low risk | Low risk | Low risk | Low risk | Low risk | Low risk | **Low risk** |
| Amoah 2003b | High risk | High risk | Low risk | High risk | High risk | Low risk | Low risk | Low risk | Low risk | Low risk | Low risk | **Moderate risk** |
| Amponsah 2019 | High risk | High risk | High risk | High risk | High risk | Low risk | Low risk | Low risk | Low risk | Low risk | Low risk | **High risk** |
| Amponsem-Boateng 2017 | Low risk | Low risk | Low risk | Low risk | High risk | Low risk | Low risk | Low risk | Low risk | Low risk | Low risk | **Low risk** |
| Anderson 2017 | High risk | High risk | High risk | High risk | High risk | Low risk | Low risk | Low risk | Low risk | Low risk | Low risk | **High risk** |
| Anto 2020 | Low risk | Low risk | Low risk | Low risk | High risk | Low risk | Low risk | Low risk | Low risk | Low risk | Low risk | **Low risk** |
| Aryeetey 2011 | Low risk | Low risk | Low risk | Low risk | High risk | Low risk | Low risk | Low risk | Low risk | Low risk | Low risk | **Low risk** |
| Atibila 2018 | Low risk | Low risk | Low risk | Low risk | High risk | Low risk | Low risk | Low risk | Low risk | Low risk | Low risk | **Low risk** |
| Atinyi 2017 | Low risk | Low risk | Low risk | Low risk | High risk | Low risk | Low risk | Low risk | Low risk | Low risk | Low risk | **Low risk** |
| Awuah 2014 | Low risk | Low risk | Low risk | Low risk | High risk | Low risk | Low risk | Low risk | Low risk | Low risk | Low risk | **Low risk** |
| Basu 2013 | Low risk | Low risk | Low risk | Low risk | High risk | Low risk | Low risk | Low risk | Low risk | Low risk | Low risk | **Low risk** |
| Bawah 2019 | High risk | High risk | Low risk | Low risk | High risk | Low risk | Low risk | Low risk | Low risk | Low risk | Low risk | **Moderate risk** |
| Bosu 2010 | Low risk | Low risk | Low risk | Low risk | High risk | Low risk | Low risk | Low risk | Low risk | Low risk | Low risk | **Low risk** |
| Burket 2006 | High risk | High risk | High risk | High risk | High risk | Low risk | Low risk | Low risk | Low risk | Low risk | Low risk | **High risk** |
| Cappuccio 2004 | Low risk | High risk | Low risk | High risk | Low risk | Low risk | Low risk | Low risk | Low risk | Low risk | Low risk | **Moderate risk** |
| Cook-Huynh 2012 | High risk | Low risk | Low risk | Low risk | High risk | Low risk | Low risk | Low risk | Low risk | Low risk | Low risk | **Moderate risk** |
| Darko 2012 | Low risk | High risk | Low risk | Low risk | High risk | Low risk | Low risk | Low risk | Low risk | Low risk | Low risk | **Low risk** |
| Donkor 2015 | Low risk | Low risk | Low risk | Low risk | High risk | Low risk | Low risk | Low risk | Low risk | Low risk | Low risk | **Low risk** |
| Dosoo 2019 | Low risk | Low risk | Low risk | Low risk | High risk | Low risk | Low risk | Low risk | Low risk | Low risk | Low risk | **Low risk** |
| Duah 2013 | Low risk | Low risk | Low risk | Low risk | High risk | Low risk | Low risk | Low risk | Low risk | Low risk | Low risk | **Low risk** |
| Duda 2007 | Low risk | High risk | Low risk | Low risk | High risk | Low risk | Low risk | Low risk | Low risk | Low risk | Low risk | **Low risk** |
| Egungwu 2015 | Low risk | Low risk | Low risk | Low risk | High risk | Low risk | Low risk | Low risk | Low risk | Low risk | Low risk | **Low risk** |
| Ellahi 2017 | Low risk | Low risk | Low risk | Low risk | High risk | Low risk | Low risk | Low risk | Low risk | Low risk | Low risk | **Low risk** |
| Escalona 2004 | High risk | High risk | High risk | High risk | High risk | Low risk | Low risk | Low risk | Low risk | Low risk | Low risk | **High risk** |
| Frimpong 2018 | High risk | High risk | High risk | High risk | High risk | Low risk | Low risk | Low risk | Low risk | Low risk | Low risk | **High risk** |
| Gato 2019 | High risk | High risk | High risk | High risk | High risk | Low risk | Low risk | Low risk | Low risk | Low risk | Low risk | **High risk** |
| Gómez-Olivé 2017 | Low risk | Low risk | Low risk | Low risk | High risk | Low risk | Low risk | Low risk | Low risk | Low risk | Low risk | **Low risk** |
| Gyamfi 2010 | Low risk | Low risk | Low risk | Low risk | High risk | Low risk | Low risk | Low risk | Low risk | Low risk | Low risk | **Low risk** |
| Gyamfi 2015 | High risk | High risk | High risk | High risk | High risk | Low risk | Low risk | Low risk | Low risk | Low risk | Low risk | **High risk** |
| Gyamfi 2018 | Low risk | Low risk | Low risk | High risk | High risk | Low risk | Low risk | Low risk | Low risk | Low risk | Low risk | **Low risk** |
| Hayibor 2010 | Low risk | Low risk | Low risk | Low risk | High risk | Low risk | Low risk | Low risk | Low risk | Low risk | Low risk | **Low risk** |
| Jaziri 2016 | Low risk | Low risk | Low risk | Low risk | High risk | Low risk | Low risk | Low risk | Low risk | Low risk | Low risk | **Low risk** |
| Kasu 2015 | Low risk | Low risk | Low risk | Low risk | High risk | Low risk | Low risk | Low risk | Low risk | Low risk | Low risk | **Low risk** |
| Kodaman 2016 | High risk | High risk | Low risk | Low risk | High risk | Low risk | Low risk | Low risk | Low risk | Low risk | Low risk | **Moderate risk** |
| Koopman 2012 | Low risk | Low risk | Low risk | Low risk | High risk | Low risk | Low risk | Low risk | Low risk | Low risk | Low risk | **Low risk** |
| Kpormegbe 2019 | High risk | Low risk | Low risk | Low risk | High risk | Low risk | Low risk | Low risk | Low risk | Low risk | Low risk | **Moderate risk** |
| Kubuga 2015 |  |  |  |  |  |  |  |  |  |  |  | **Can't tell** |
| Kunutsor 2009 | Low risk | Low risk | Low risk | Low risk | High risk | Low risk | Low risk | Low risk | Low risk | Low risk | Low risk | **Low risk** |
| Lamptey 2017 | Low risk | Low risk | Low risk | Low risk | High risk | Low risk | Low risk | Low risk | Low risk | Low risk | Low risk | **Low risk** |
| Mensah 2013 | High risk | Low risk | Low risk | High risk | High risk | Low risk | Low risk | Low risk | Low risk | Low risk | Low risk | **Moderate risk** |
| Mensa-Wilmot 2003 | Low risk | Low risk | Low risk | Low risk | High risk | Low risk | Low risk | Low risk | Low risk | Low risk | Low risk | **Low risk** |
| Menyanu 2017 | Low risk | Low risk | Low risk | Low risk | High risk | Low risk | Low risk | Low risk | Low risk | Low risk | Low risk | **Low risk** |
| Mohammed 2016 | Low risk | Low risk | Low risk | Low risk | High risk | Low risk | Low risk | Low risk | Low risk | Low risk | Low risk | **Low risk** |
| Murray 2018 | High risk | High risk | High risk | High risk | High risk | Low risk | Low risk | Low risk | Low risk | Low risk | Low risk | **High risk** |
| Newlove 2011 | Low risk | Low risk | Low risk | Low risk | High risk | Low risk | Low risk | Low risk | Low risk | Low risk | Low risk | **Low risk** |
| Nuertey 2017 | High risk | Low risk | Low risk | Low risk | High risk | Low risk | Low risk | Low risk | Low risk | Low risk | Low risk | **Moderate risk** |
| Nunoo 2018 | High risk | High risk | High risk | High risk | High risk | Low risk | Low risk | Low risk | Low risk | Low risk | Low risk | **High risk** |
| Nyarko 2018 | Low risk | Low risk | Low risk | Low risk | High risk | Low risk | Low risk | Low risk | Low risk | Low risk | Low risk | **Low risk** |
| Obirikorang 2015 | Low risk | Low risk | Low risk | Low risk | High risk | Low risk | Low risk | Low risk | Low risk | Low risk | Low risk | **Low risk** |
| Ofosuhene 2020 | Low risk | Low risk | Low risk | Low risk | High risk | Low risk | Low risk | Low risk | Low risk | Low risk | Low risk | **Low risk** |
| Osei-Yeboah 2018 | High risk | Low risk | High risk | Low risk | High risk | Low risk | Low risk | Low risk | Low risk | Low risk | Low risk | **Moderate risk** |
| Osman 2017 | High risk | High risk | Low risk | Low risk | High risk | Low risk | Low risk | Low risk | Low risk | Low risk | Low risk | **High risk** |
| Owiredu 2008 | High risk | Low risk | Low risk | High risk | High risk | Low risk | Low risk | Low risk | Low risk | Low risk | Low risk | **Moderate risk** |
| Owiredu 2011 | High risk | Low risk | High risk | High risk | High risk | Low risk | Low risk | Low risk | Low risk | Low risk | Low risk | **High risk** |
| Owusu-Sekyere 2018 | High risk | High risk | Low risk | High risk | High risk | Low risk | Low risk | Low risk | Low risk | Low risk | Low risk | **Moderate risk** |
| Pobee 2006 | Low risk | High risk | High risk | High risk | High risk | Low risk | Low risk | Low risk | Low risk | Low risk | Low risk | **Low risk** |
| Pobee 2013 | High risk | Low risk | Low risk | Low risk | High risk | Low risk | Low risk | Low risk | Low risk | Low risk | Low risk | **Moderate risk** |
| Rajaee 2015 | Low risk | Low risk | Low risk | Low risk | High risk | Low risk | Low risk | Low risk | Low risk | Low risk | Low risk | **Low risk** |
| Sanuade 2018 | High risk | Low risk | Low risk | Low risk | High risk | Low risk | Low risk | Low risk | Low risk | Low risk | Low risk | **Moderate risk** |
| Sarfo-Kantanka 2014 | High risk | High risk | High risk | Low risk | High risk | Low risk | Low risk | Low risk | Low risk | Low risk | Low risk | **High risk** |
| Sarkodie 2018 | Low risk | Low risk | Low risk | Low risk | High risk | Low risk | Low risk | Low risk | Low risk | Low risk | Low risk | **Low risk** |
| Setorglo 2019 | Low risk | Low risk | Low risk | Low risk | High risk | Low risk | Low risk | Low risk | Low risk | Low risk | Low risk | **Low risk** |
| Shaidah 2016 | High risk | High risk | Low risk | High risk | High risk | Low risk | Low risk | Low risk | Low risk | Low risk | Low risk | **High risk** |
| Solomon 2017 | Low risk | Low risk | Low risk | Low risk | High risk | Low risk | Low risk | Low risk | Low risk | Low risk | Low risk | **Low risk** |
| Taylor 2015 | High risk | High risk | High risk | High risk | High risk | Low risk | Low risk | Low risk | Low risk | Low risk | Low risk | **High risk** |
| Vuvor 2011 | Low risk | Low risk | Low risk | Low risk | High risk | Low risk | Low risk | Low risk | Low risk | Low risk | Low risk | **Low risk** |
| Vuvor 2016 | High risk | High risk | High risk | High risk | High risk | Low risk | Low risk | Low risk | Low risk | Low risk | Low risk | **High risk** |
| Vuvor 2017 | High risk | High risk | High risk | High risk | High risk | Low risk | Low risk | Low risk | Low risk | Low risk | Low risk | **High risk** |
| Yakong 2015 | High risk | Low risk | Low risk | Low risk | High risk | Low risk | Low risk | Low risk | Low risk | Low risk | Low risk | **Low risk** |
| Yeboah 2015 | High risk | High risk | High risk | High risk | High risk | Low risk | Low risk | Low risk | Low risk | Low risk | Low risk | **High risk** |
