# Supplementary figures and images for: Prevalence, awareness and control of hypertension in Ghana: A systematic review and meta-analysis
Source: PLoS One. 2021 Mar 5;16(3):e0248137. doi: 10.1371/journal.pone.0248137 (PMC7935309; doi:10.1371/journal.pone.0248137)

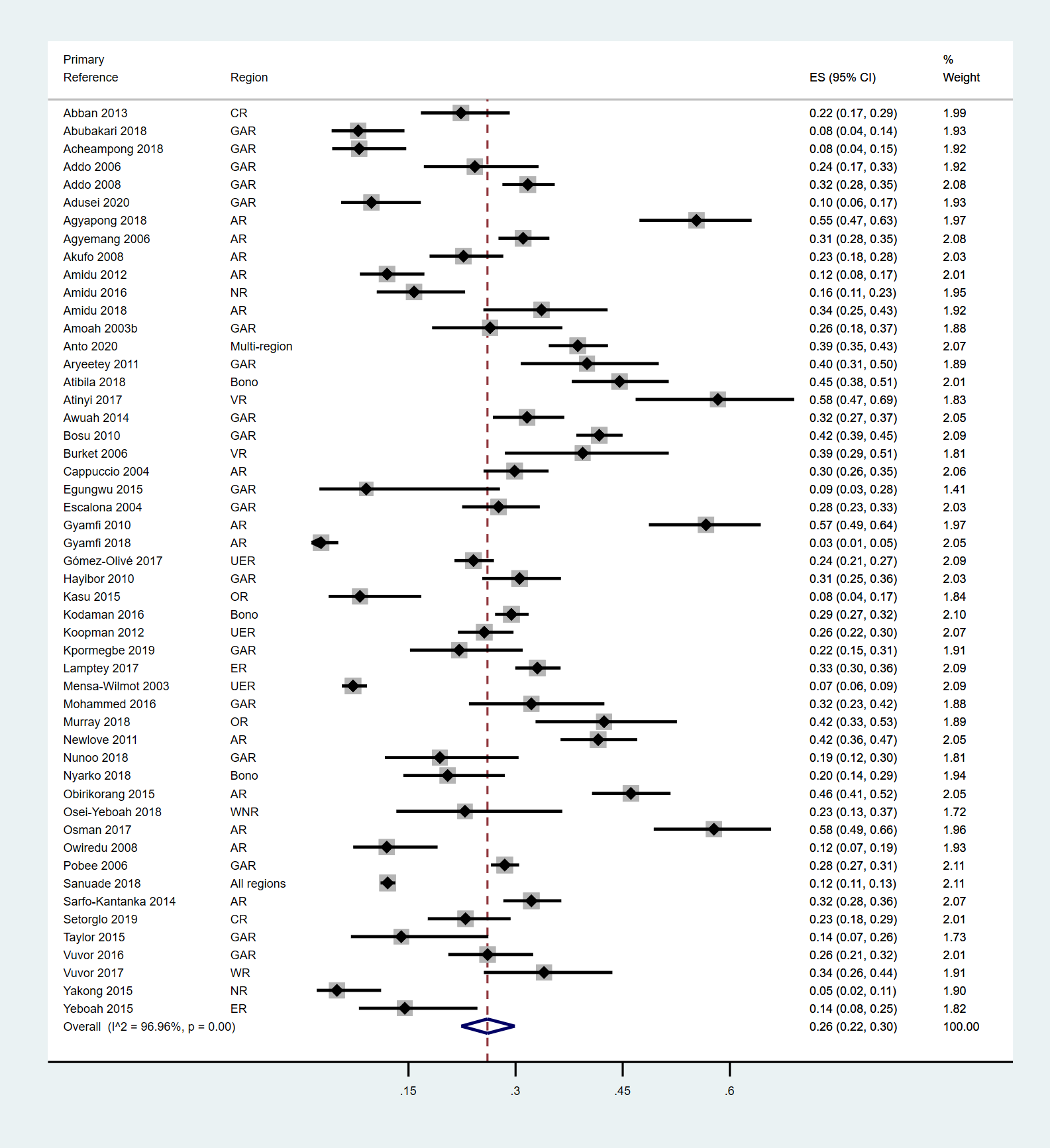

Supplement: S1 Fig — (TIF) [file pone.0248137.s005.tif]

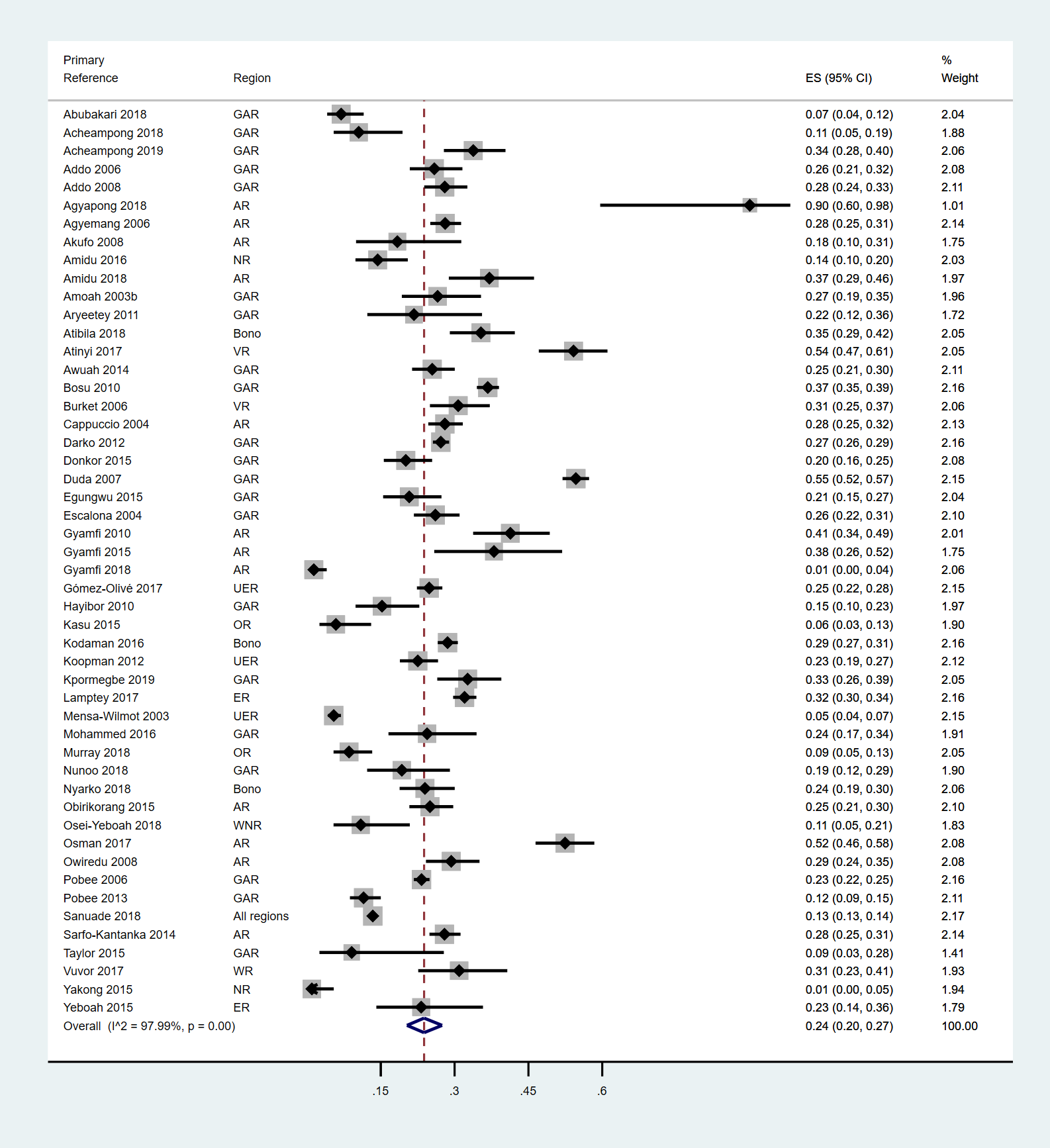

Supplement: S2 Fig — (TIF) [file pone.0248137.s006.tif]

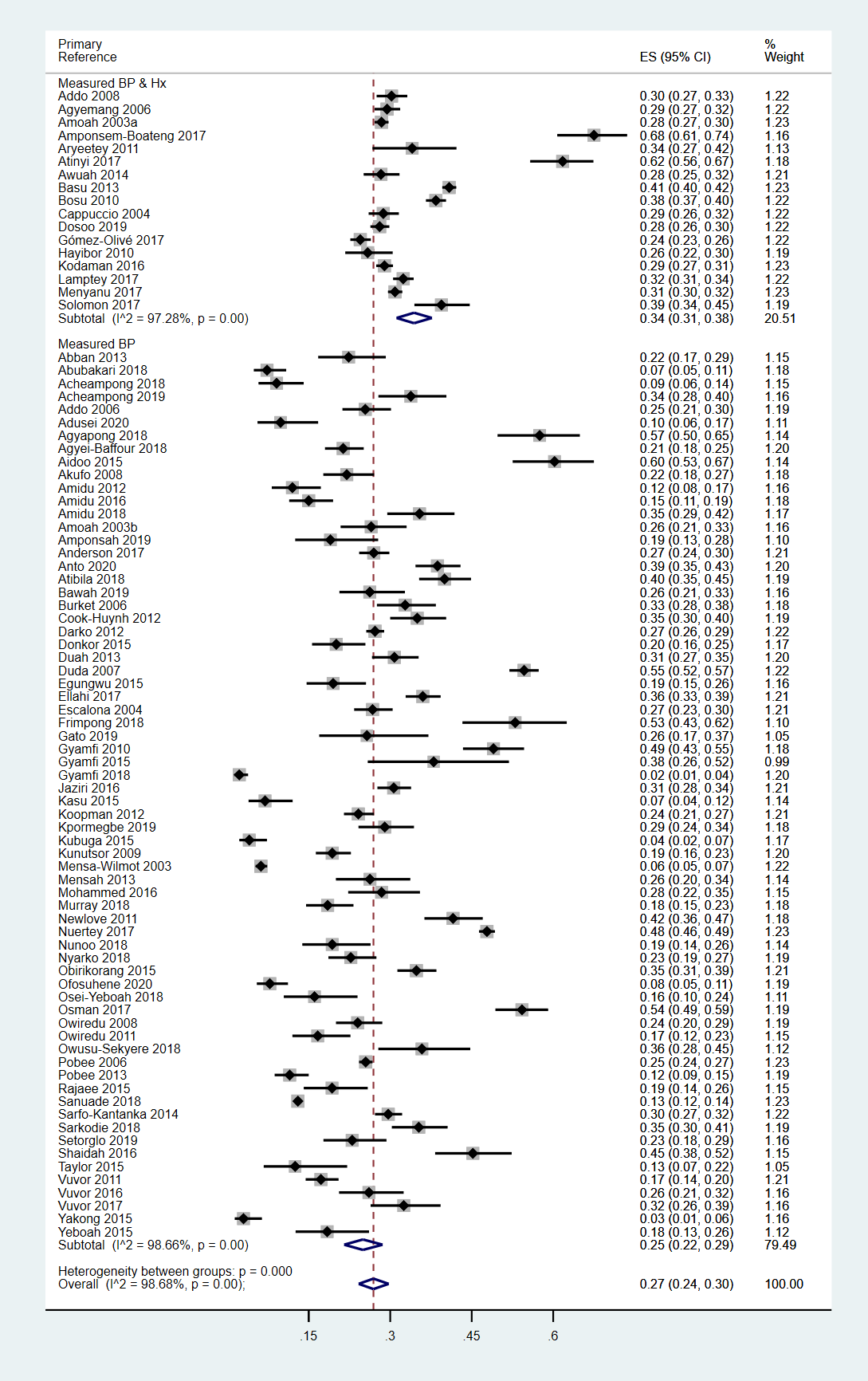

Supplement: S3 Fig — (TIF) [file pone.0248137.s007.tif]

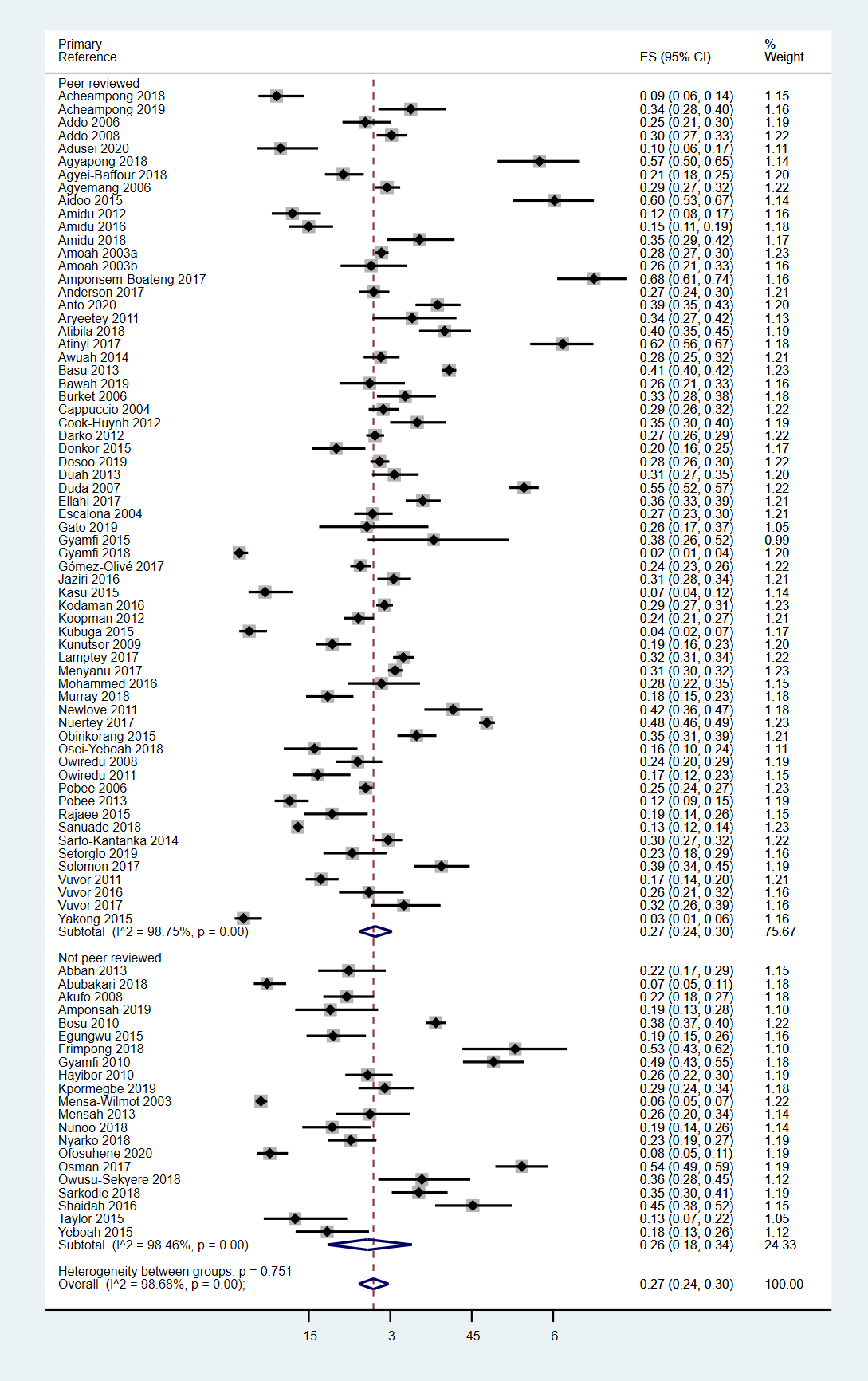

Supplement: S4 Fig — (TIF) [file pone.0248137.s008.tif]

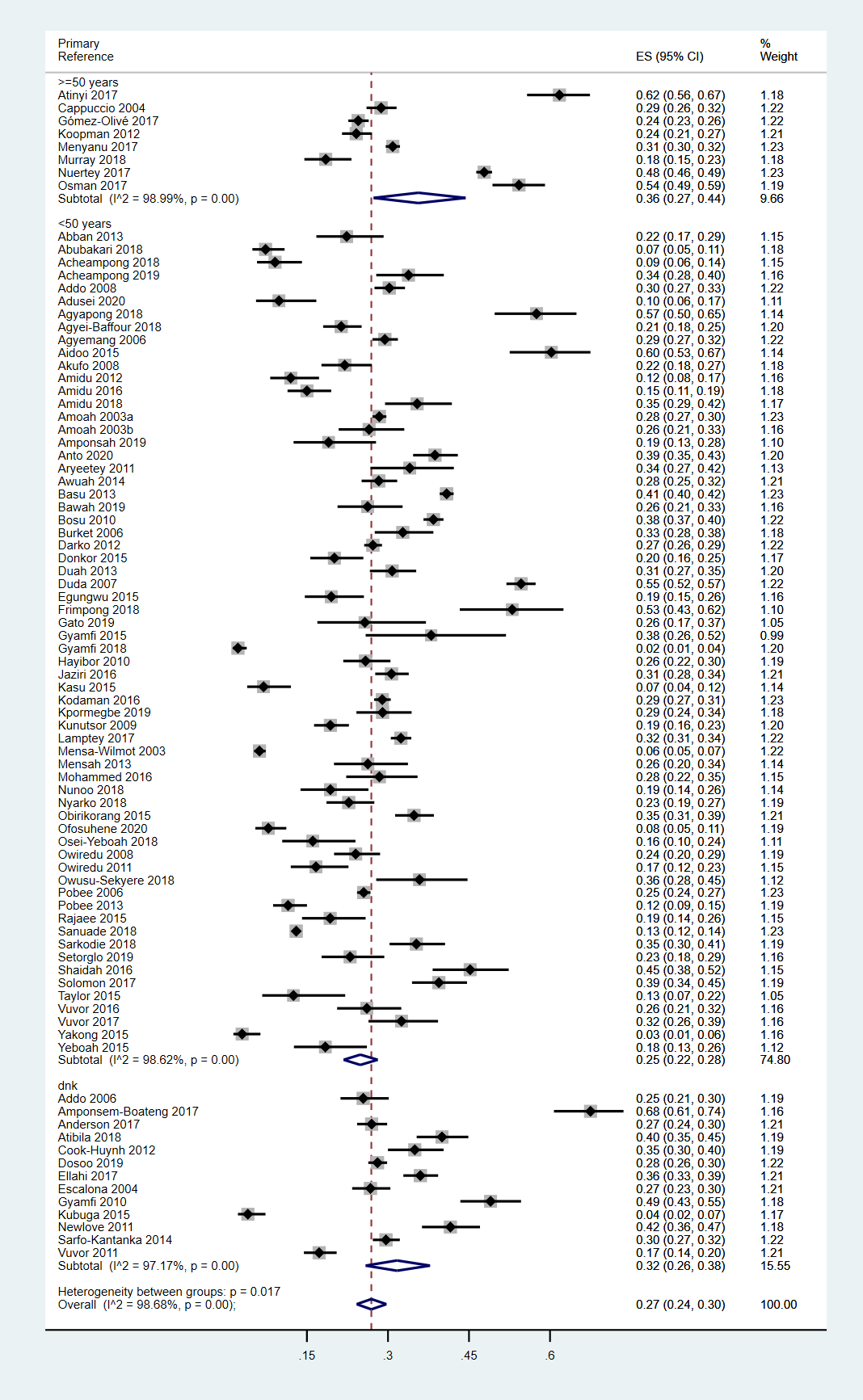

Supplement: S5 Fig — (TIF) [file pone.0248137.s009.tif]

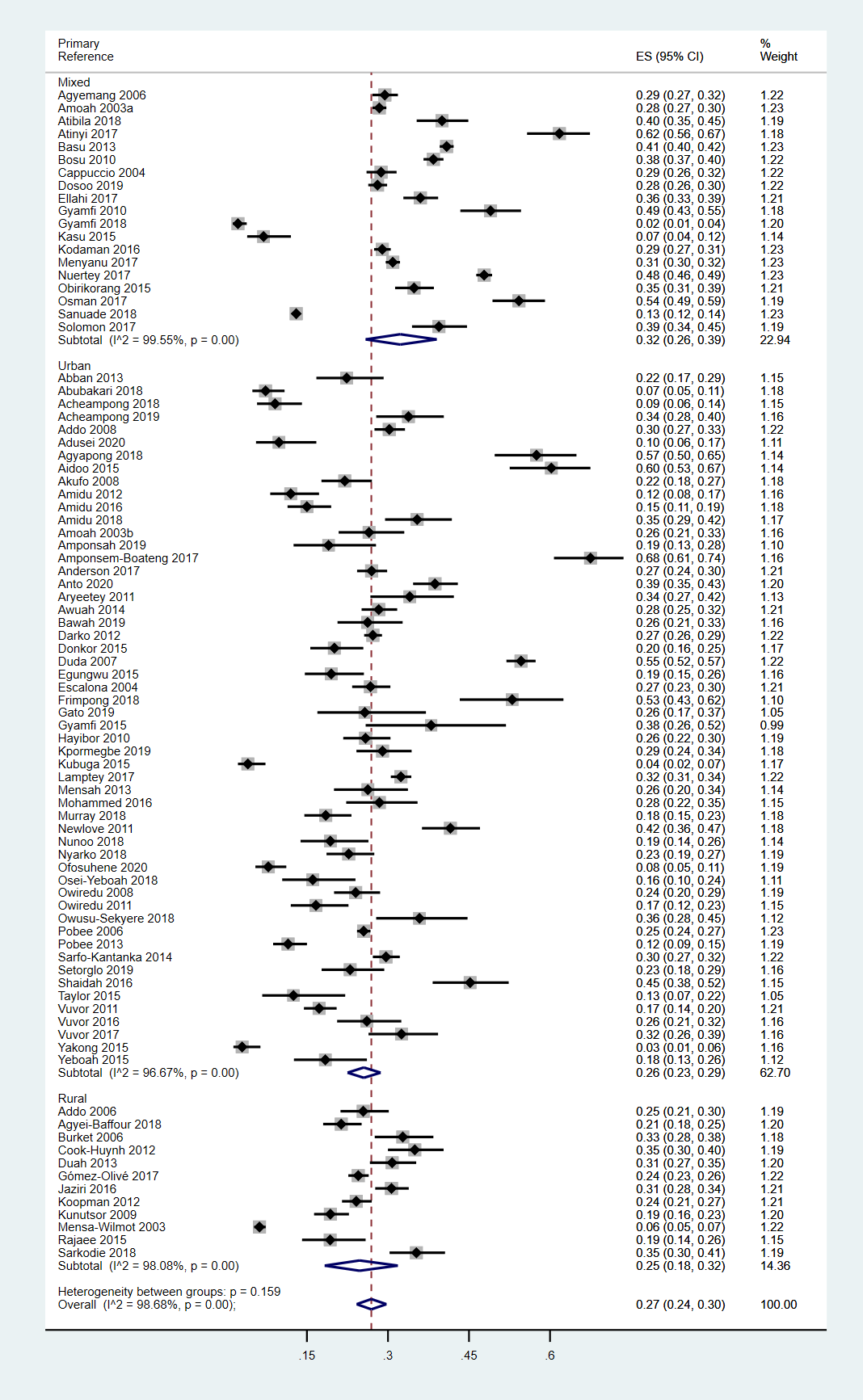

Supplement: S6 Fig — (TIF) [file pone.0248137.s010.tif]

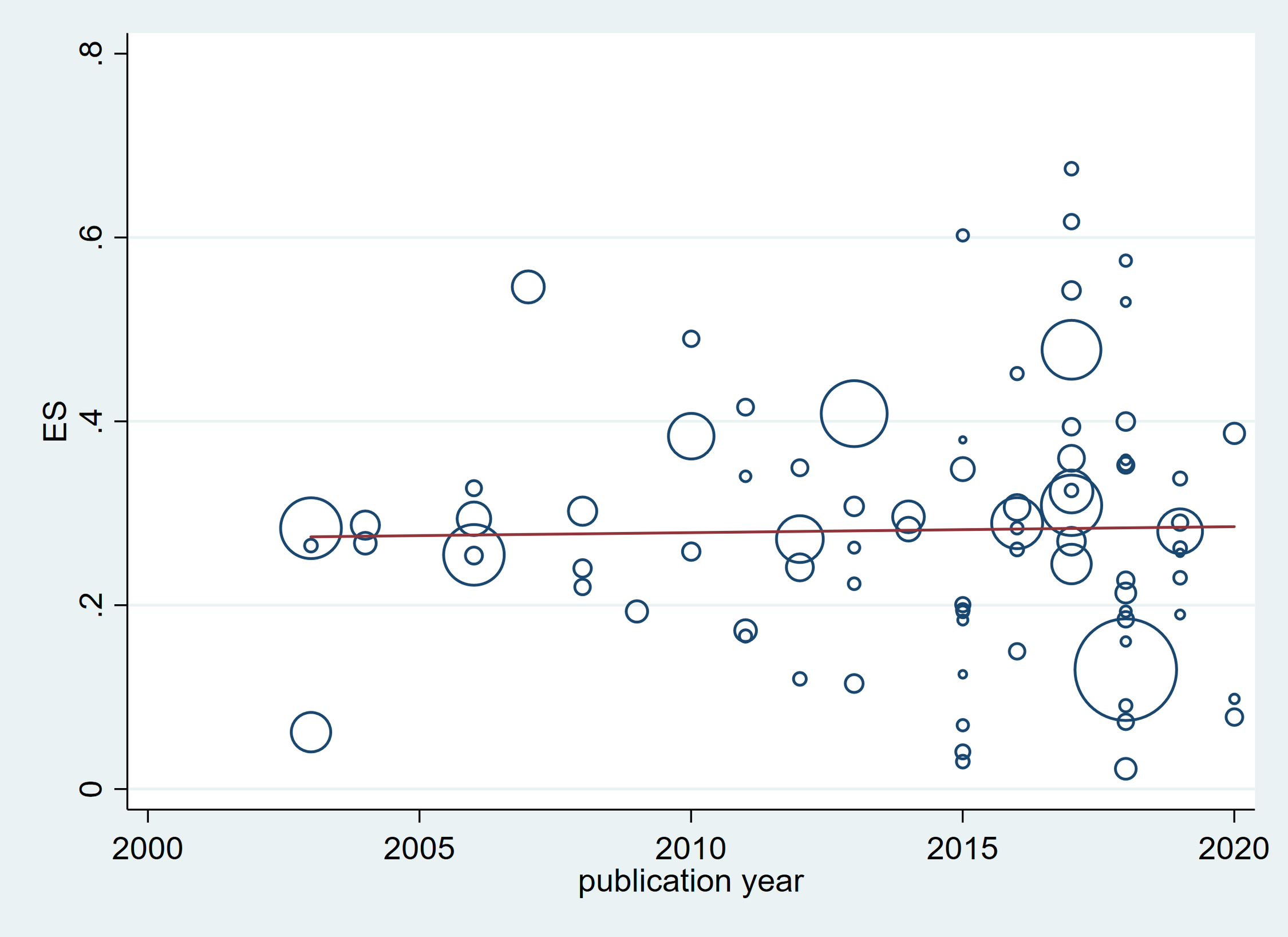

Supplement: S7 Fig — (TIF) [file pone.0248137.s011.tif]

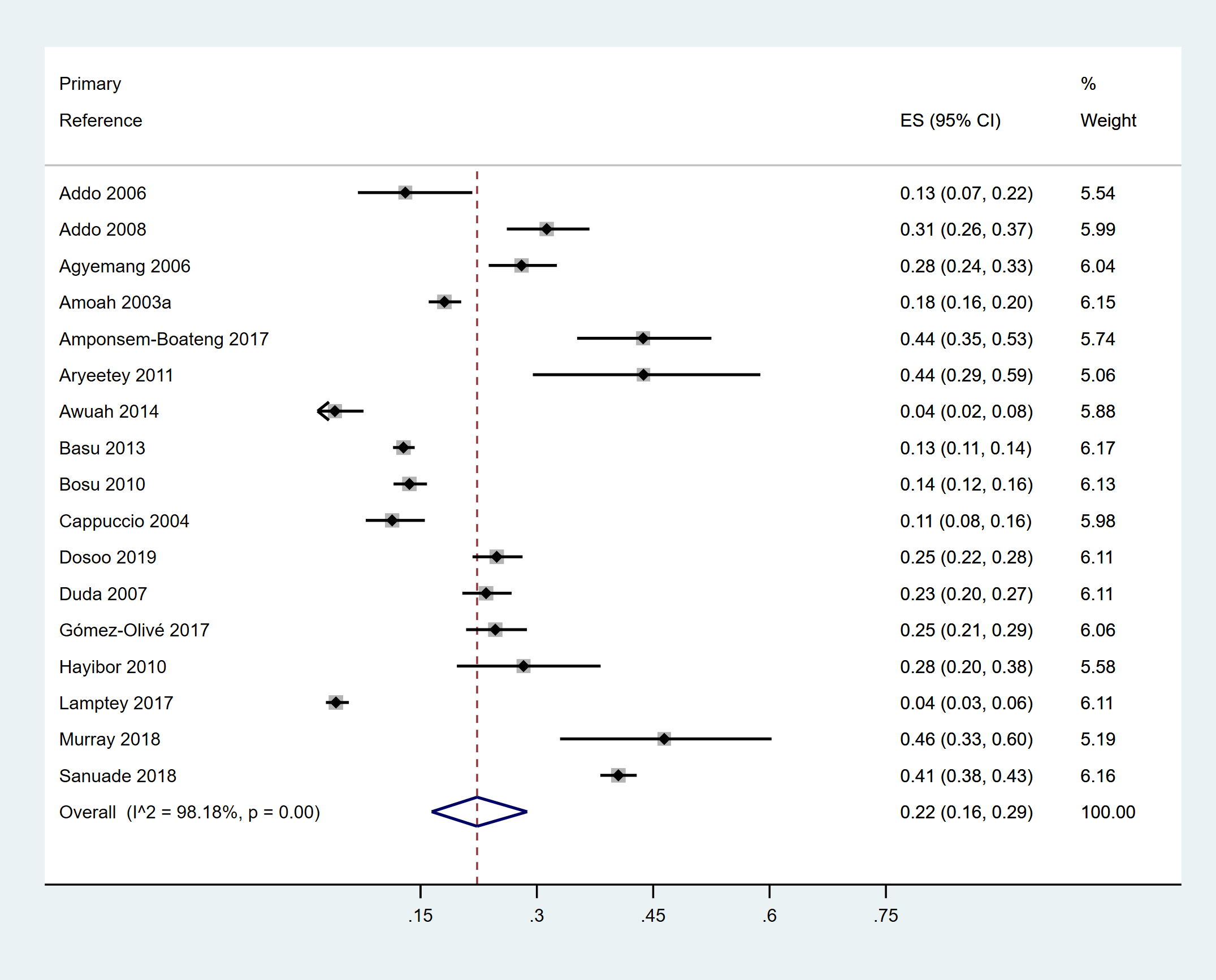

Supplement: S8 Fig — (TIF) [file pone.0248137.s012.tif]

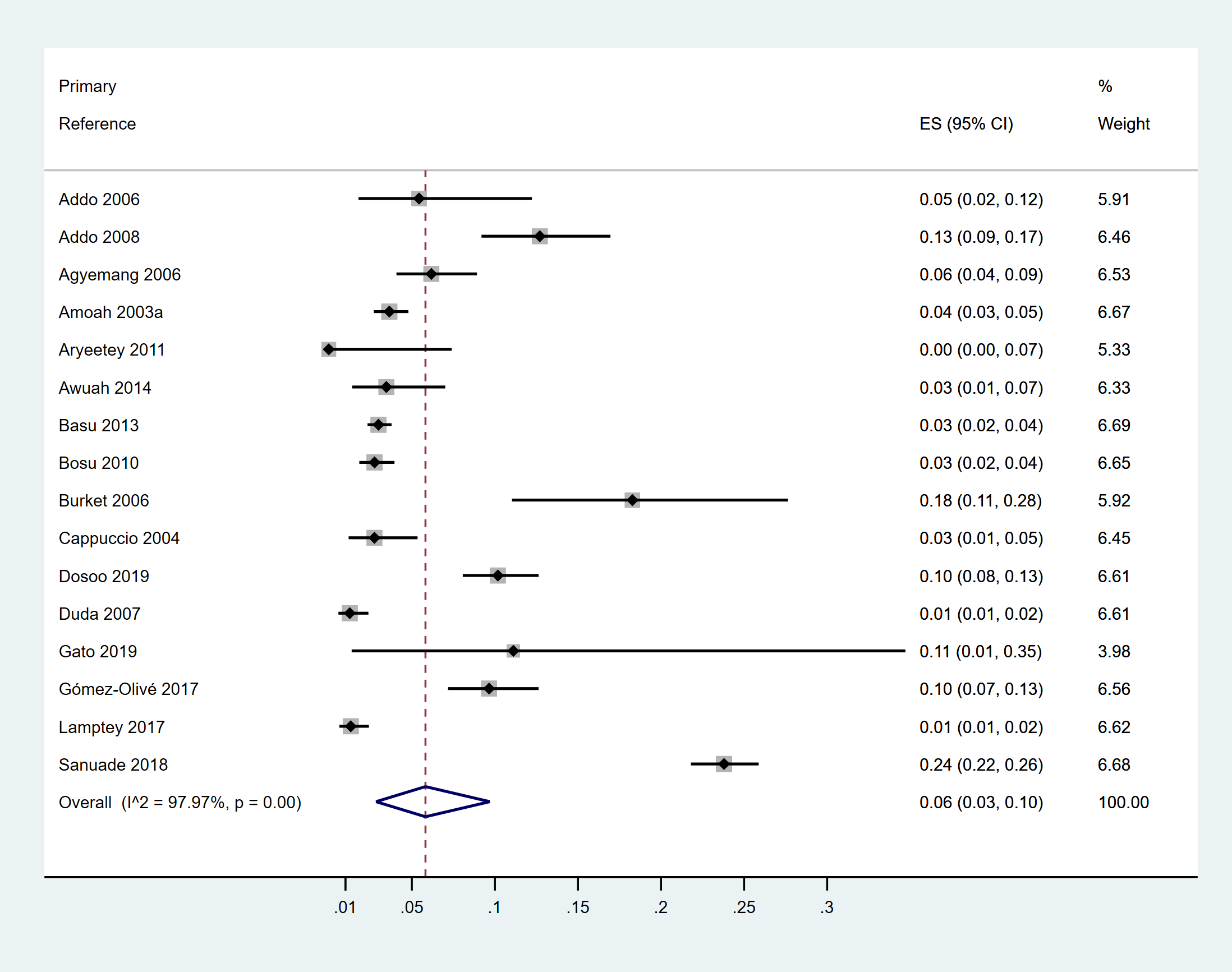

Supplement: S9 Fig — (TIF) [file pone.0248137.s013.tif]

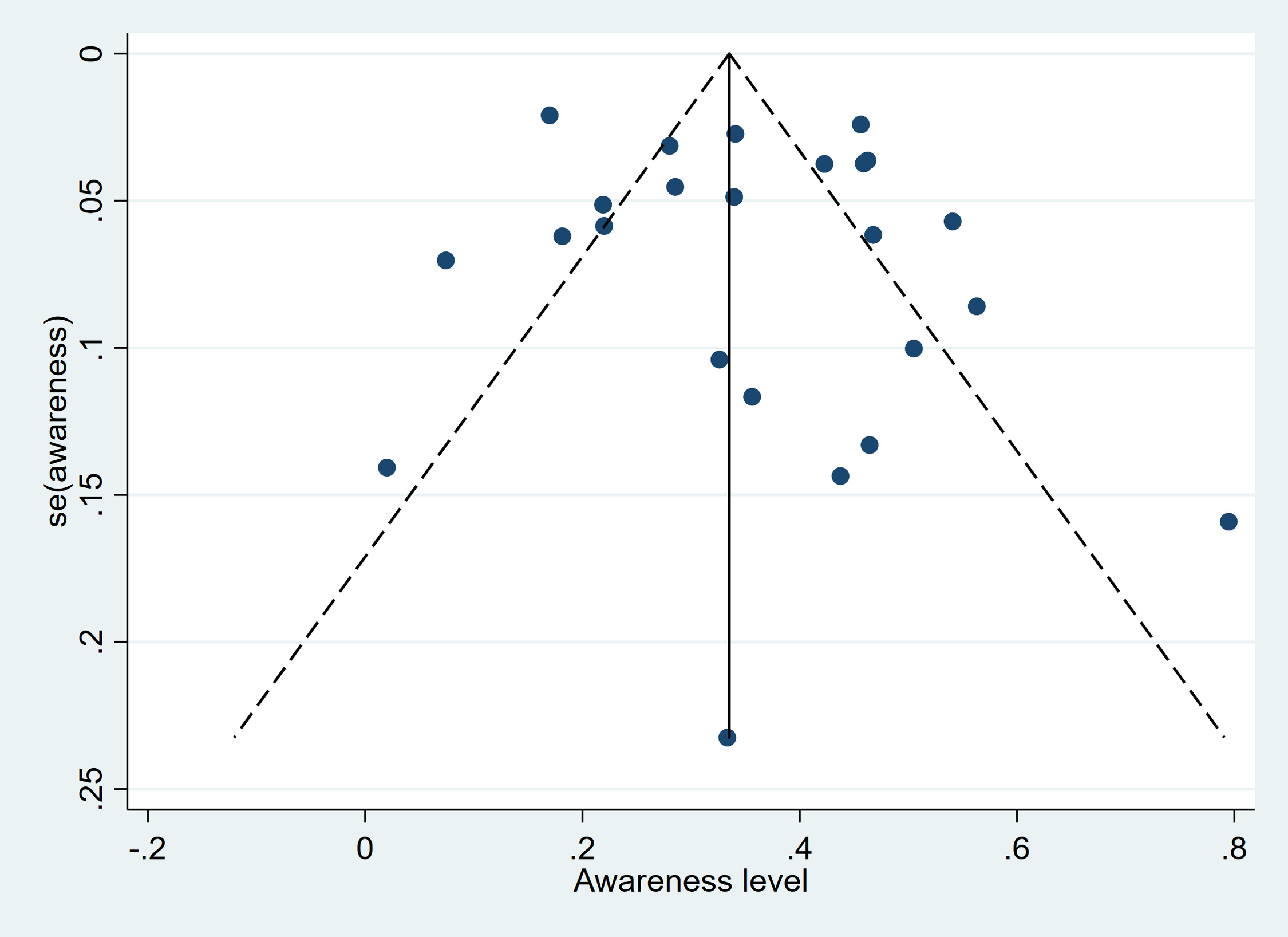

Supplement: S10 Fig — (TIF) [file pone.0248137.s014.tif]

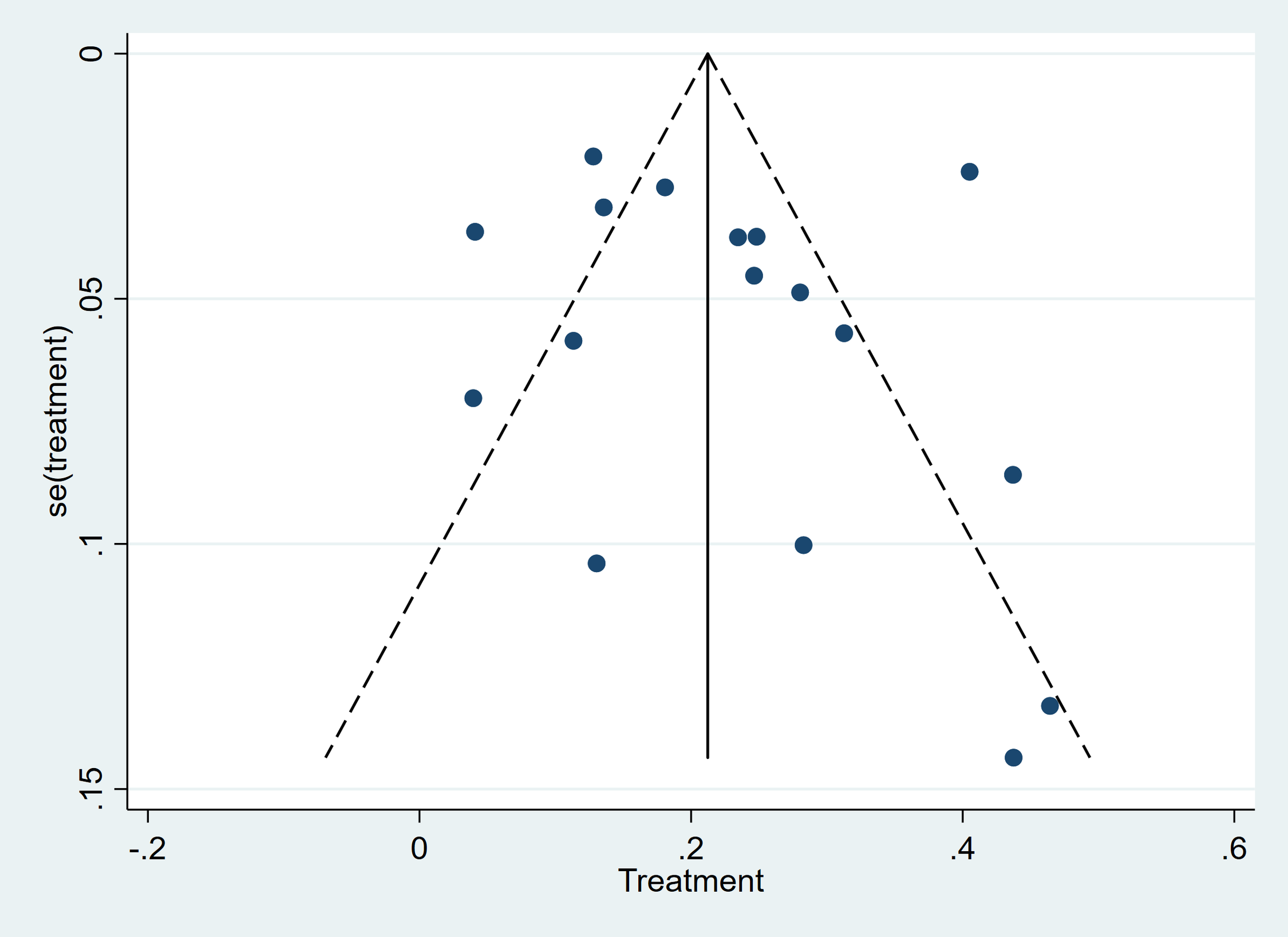

Supplement: S11 Fig — (TIF) [file pone.0248137.s015.tif]

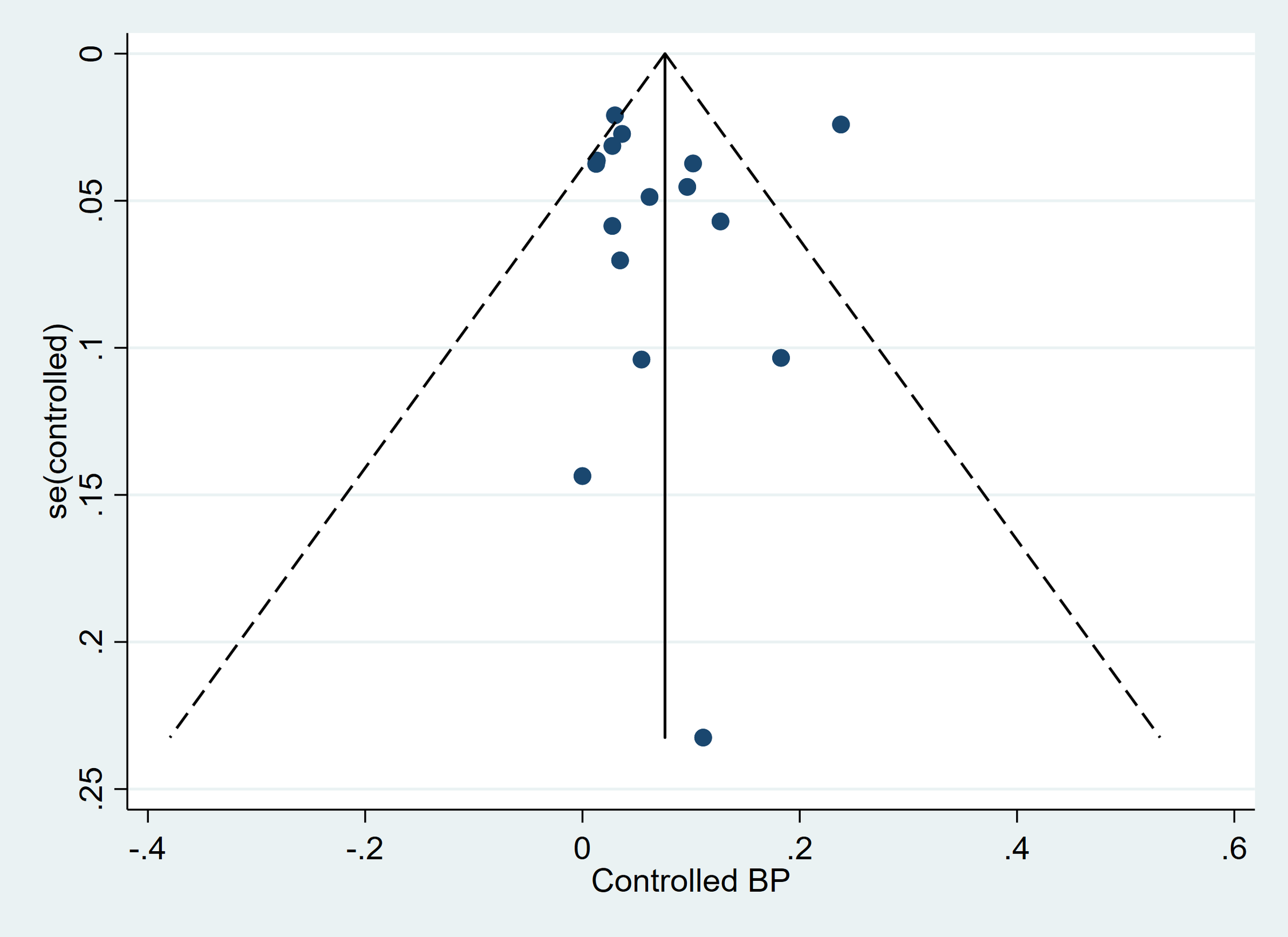

Supplement: S12 Fig — (TIF) [file pone.0248137.s016.tif]
